# Superatom Distortion Induces Triferroicity and Spin Splitting in Two‐Dimensional Antiferromagnets

**DOI:** 10.1002/advs.202600005

**Published:** 2026-04-07

**Authors:** Zhen Gao, Fengxian Ma, Guoping Gao, Weizhen Meng, Ying Liu, Yandong Ma, Yalong Jiao

**Affiliations:** ^1^ College of Physics Hebei Key Laboratory of Photophysics Research and Application Hebei Normal University Shijiazhuang China; ^2^ MOE Key Laboratory for Non‐equilibrium Synthesis and Modulation of Condensed Matter Shaanxi Province Key Laboratory of Advanced Functional Materials and Mesoscopic Physics School of Physics Xi'an Jiaotong University Xi'an Shaanxi China; ^3^ School of Physics State Key Laboratory of Crystal Materials Shandong University Jinan China

**Keywords:** first‐principles calculations, multiferroics, spin splitting antiferromagnets, superatom, two‐dimensional materials

## Abstract

Triferroicity in two‐dimensional (2D) materials provides an exceptional platform for multifunctional devices, yet combining a spin‐splitting antiferromagnetic (AFM) state with ferroelectricity and ferroelasticity remains a formidable challenge. Here, we propose a general strategy to achieve concurrent ferroelectricity, AFM spin splitting, and ferroelasticity by incorporating superatoms into 2D lattices. We show that strong *p‐d* orbital hybridization induces spontaneous symmetry‐lowering distortions of superatoms, breaking inversion symmetry and stabilizing magnetic ordering. This mechanism enables the coexistence of these three ferroic orders—an effect that is difficult to achieve with conventional atomic building blocks. Using first‐principles calculations, we identify the NbB_12_H_6_ monolayer as a representative example, in which the Jahn‐Teller effect drives the structural distortion. Crucially, reversing the ferroelectric polarization in the NbB_12_H_6_ leads to a deterministic reversal of the AFM spin splitting, demonstrating robust magnetoelectric coupling. Moreover, this superatom‐based multiferroic framework can be readily extended to a broad class of materials through the incorporation of alternative metals or superatomic motifs. Our findings establish superatom assembly as a powerful paradigm for designing 2D multiferroics with controllable spin degrees of freedom.

## Graphical abstract

The incorporation of superatoms into a 2D square lattice induces symmetry breaking, thereby enabling concurrent coupling among magnetism, ferroelectricity, and ferroelasticity. This strategy achieves triferroic behavior—characterized by spin‐split antiferromagnetic ground states—and offers a viable pathway toward energy‐efficient spintronic devices.

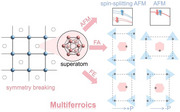

## 1 Introduction

Ferroic materials constitute a cornerstone of functional condensed matter physics, characterized by switchable order parameters—such as electric polarization, spin polarization, and lattice strain—in response to conjugate external stimuli [1]. This intrinsic controllability gives rise to rich physical functionalities and underpins a broad range of applications, including nonvolatile memory devices, actuators, and sensors [2, 3, 4, 5, 6, 7].

Recently, the emergence of altermagnets (AMs) [8, 9, 10], or more broadly antiferromagnets with spin splitting (AFM‐ss), has profoundly reshaped the classification of magnetism and expanded the scope of multiferroic research. By combining the key advantages of ferromagnets (sizable spin splitting) and conventional antiferromagnets (zero net magnetization), AFM‐ss systems offer a unique platform for coupling spin degrees of freedom with other ferroic orders [11, 12]. Integrating AFM‐ss phases with additional ferroic channels is expected to give rise to unconventional physical phenomena that are absent in traditional multiferroics. For example, coupling the AFM‐ss state with ferroelectricity (FE) enables electrically switchable spin states [13], strong FE‐AM coupling in low dimensions [14], electrically switchable magnonic properties [15], and symmetry‐mediated spin‐ferroelectric locking [16]. Meanwhile, coupling with ferroelasticity (FA) allows for crystal‐orientation‐driven modulation of AFM spin splitting [17]. Despite this progress, current research has largely been confined to the coexistence of only two ferroic orders [18, 19, 20]. The simultaneous integration of FE, FA, and AFM‐ss (i.e., triferroicity) within a single two‐dimensional (2D) lattice remains a formidable challenge. This difficulty arises from the stringent and often conflicting symmetry requirements and electronic mechanisms governing these distinct order parameters. Consequently, a general strategy for realizing 2D triferroics hosting AFM‐ss states is urgently needed to advance multiferroic‐based device concepts.

In 2D systems, the coexistence of both FE and AFM‐ss requires the breaking of inversion symmetry, while FA demands a low‐symmetry lattice capable of sustaining reversible structural deformation. Satisfying these criteria simultaneously necessitates substantial yet controllable lattice distortions, which are difficult to achieve using conventional atomic building blocks. In this context, superatoms, atomically precise clusters that mimic the electronic behavior of single elements, offer a promising solution. Unlike isolated atoms, superatoms can possess finite‐volume, cage‐like architectures that sustain intrinsic distortion, polarity, and delocalized electrons [21, 22]. These electrons form superatomic orbitals (SAOs) that can hybridize with neighboring atomic orbitals (AOs), thereby lowering the symmetry of the superatom and inducing cluster‐level polarizability. The resulting polarized superatoms generate additional local electrostatic fields, which drive atomic displacements in the surrounding lattice and break inversion symmetry. As a result, the key structural and symmetry requirements for realizing multiple ferroic orders can be naturally satisfied by embedding superatoms into 2D lattices.

In this work, we propose a general design principle for realizing 2D triferroic AFM‐ss state via superatom engineering. We adopt the 2D square lattice as a model system in view of its high structural symmetry and the availability of multiple symmetry‐breaking pathways. In its high symmetry form, the square lattice exhibits fourfold rotational symmetry (*C_4_*), which can be selectively reduced by lattice distortions, atomic off‐centering, or spin ordering. This inherent symmetry tunability can provide a natural platform for the coexistence and mutual coupling of multiple ferroic orders. To establish a baseline, we first examine an experimentally realized checkerboard lattice and demonstrate that conventional atomic constituents are insufficient to induce the lattice distortions required for simultaneous ferroic ordering. In contrast, we show that introducing a superatom can effectively break inversion symmetry through strong *p*‐*d* orbital hybridization between the superatom and neighboring transition‐metal atoms. This interaction drives pronounced local distortions and enables the emergence of coupled ferroic states. Based on first‐principles calculations combined with high‐throughput screening across transition‐metal elements, we identify the NbB_12_H_6_ monolayer—derived from the B_12_H_12_ superatom—as an ideal candidate for hosting a 2D triferroic phase with AFM‐ss. We systematically elucidate the microscopic origins of the FE polarization, AFM‐ss states, and the FA phase transition in the NbB_12_H_6_ monolayer. Furthermore, through comprehensive materials screening, we demonstrate that the emergence of multiferroic phases in 2D systems can be generalized to a broader class of systems by varying the metal/superatom constituents.

## 2 Results and Discussion

### 2.1 A Checkerboard Lattice Model

**FIGURE 1:**
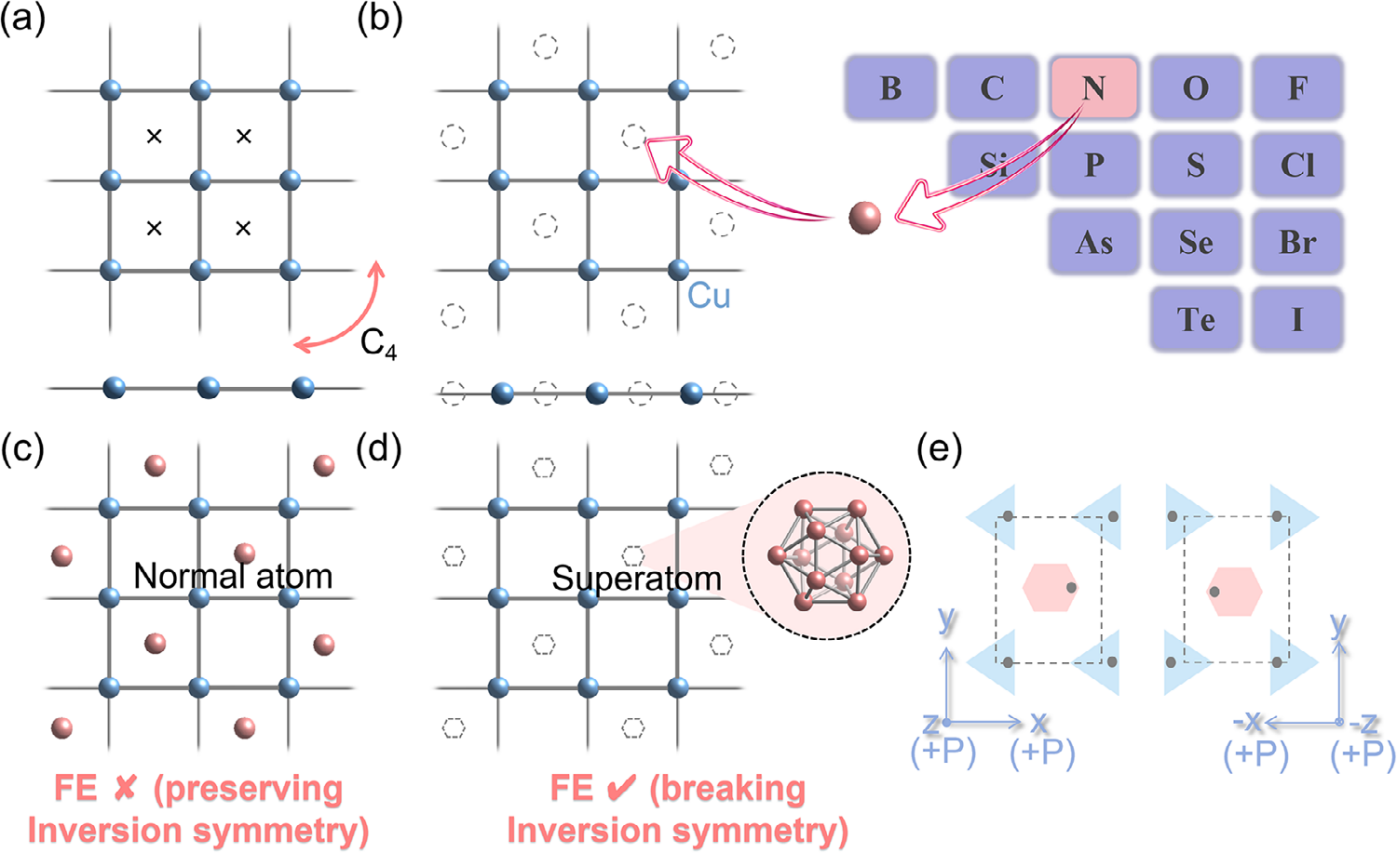
(a) An ideal two‐dimensional square lattice, with the inversion center marked by “×”. (b) A checkerboard lattice capable of accommodating the 14 selected main‐group atoms at the central sites (dashed circles). (c) Insertion of a conventional atom at the square center preserves inversion symmetry and therefore does not induce ferroic orders (e.g., FE). (d) In contrast, insertion of a superatom at the same position breaks inversion symmetry. (e) Schematic illustration of ferroelectric polarization switching, where triangles and hexagons denote metal atoms and superatoms, respectively. Gray dots indicate the displacement of charge centers. The directions of spontaneous polarization are along the x‐ and z‐directions.

In principle, lattice distortion is a prerequisite for stabilizing a 2D triferroic phase, as it provides the necessary symmetry breaking for coupled ferroic orders. To test the feasibility of this using standard building blocks, we first examine a conventional atomic square lattice. We selected the square lattice as a prototype because it combines structural simplicity with a favorable symmetry framework: its centrosymmetric parent phase provides clear insertion sites, and symmetry lowering from this lattice can naturally support both FE and FA order. In its undistorted high‐symmetry state (typically point group *D_4h_*), the lattice possesses a fourfold rotation axis (*C_4_*), mirror planes, and an inversion center (Figure 1a). Due to the presence of inversion symmetry, the polarization vector transforms as a nonpolar representation under *C_4v_*, the formation of a spontaneous electric polarization is strictly forbidden. Consequently, neither FE nor magnetoelectric coupling can emerge in this pristine lattice configuration.

One potential route to induce symmetry breaking is the introduction of foreign atoms into the lattice. However, when conventional atoms are incorporated—typically occupying the high‐symmetry centers of square plaquettes—the overall symmetry of the lattice is typically preserved. A representative example is the experimentally realized checkerboard Cu_2_N monolayer [23], where nitrogen atoms reside at the fourfold hollow sites of the Cu square lattice (Figure 1b). Despite the additional basis atoms, the system remains invariant under the high‐symmetry operations, and the lattice distortion modes transforming as polar irreducible representations remain inactive. Replacing nitrogen atoms with other main‐group elements yields an analogous outcome: both the site symmetry and space‐group symmetry are robustly maintained, preventing the activation of any FE or FA order parameter. This symmetry rigidity can be traced to the intrinsic electronic character of conventional atoms. Their nearly spherical and localized charge distributions couple weakly to lattice degrees of freedom, failing to generate long‐range electrostatic fields necessary to condense a symmetry‐lowering phonon mode. As a result, the lattice does not undergo a spontaneous structural transition to a polar subgroup (Figure 1c). This symmetry‐imposed limitation highlights the fundamental inadequacy of utilizing standard atomic building blocks to engineer triferroic phases in square‐lattice systems.

This symmetry impasse can be effectively surmounted by introducing superatoms into the lattice. Unlike ordinary atoms, which are well‐approximated as rigid, point‐like entities with nearly spherical charge distributions, superatoms possess a finite volume and complex multicenter bonding, endowing them with internal structural flexibility and additional symmetry degrees of freedom. When embedded in a square lattice, the superatom exhibits strong electronic and elastic coupling with its neighboring atoms, activating distinct symmetry‐lowering mechanisms: (i) The internal cage of the superatom can undergo anisotropic deformation under directional bonding, reducing its local symmetry; (ii) The delocalized electronic states exhibit large polarizability, allowing the superatom to respond nonlinearly to local electrostatic fields; and (iii) Through strain–charge coupling, the distorted superatom exerts feedback forces on the surrounding lattice, displacing nearby atoms and driving the system into a lower‐symmetry subgroup that lacks inversion symmetry (Figure 1d). Collectively, these effects enable the emergence of ferroic orders in an otherwise centrosymmetric 2D square lattice, establishing superatoms as effective symmetry‐active building blocks for engineering multiferroicity (Figure 1e). Notice that, although the square lattice is used as the prototype host, the underlying mechanism is not specific to this geometry. In general, any 2D lattice that can accommodate a superatom in a commensurate parent structure and support cooperative symmetry lowering toward a polar and FA phase may serve as a suitable platform for superatom‐based multiferroicity.

Superatoms are generally larger than conventional atoms; their incorporation into a checkerboard lattice is governed primarily by crystallographic compatibility and symmetry adaptability rather than size alone. A suitable host lattice should provide a commensurate centrosymmetric parent framework with well‐defined high‐symmetry insertion sites, while the superatom should possess sufficient internal deformability and anisotropic bonding capability. In addition, the host‐superatom coupling must be strong enough to drive a symmetry‐lowering distortion without compromising structural stability. These considerations can also be regarded as general design rules for superatom‐hostable monolayers.

### 2.2 Material Realization

To validate the feasibility of our design strategy, we employ a representative system based on the *closo*‐dodecaborate superatom (icosahedral B_12_H_12_), a well‐established borane cluster that has been experimentally synthesized since the 1960s [24]. When introduced at the center of a square lattice, the superatom induces pronounced local symmetry breaking in the Cu framework (Figure 1d; Figure S1a), driving a polar lattice distortion. This results in a substantial spontaneous polarization of up to 1.61 × 10^−10^ C/m. However, despite the successful symmetry reduction, the phonon spectrum reveals the presence of imaginary modes (Figure S1b), indicating dynamic instability. This finding confirms the symmetry‐breaking capability of the superatom but necessitates the exploration of alternative, dynamically stable superatom‐based 2D architectures.

**FIGURE 2:**
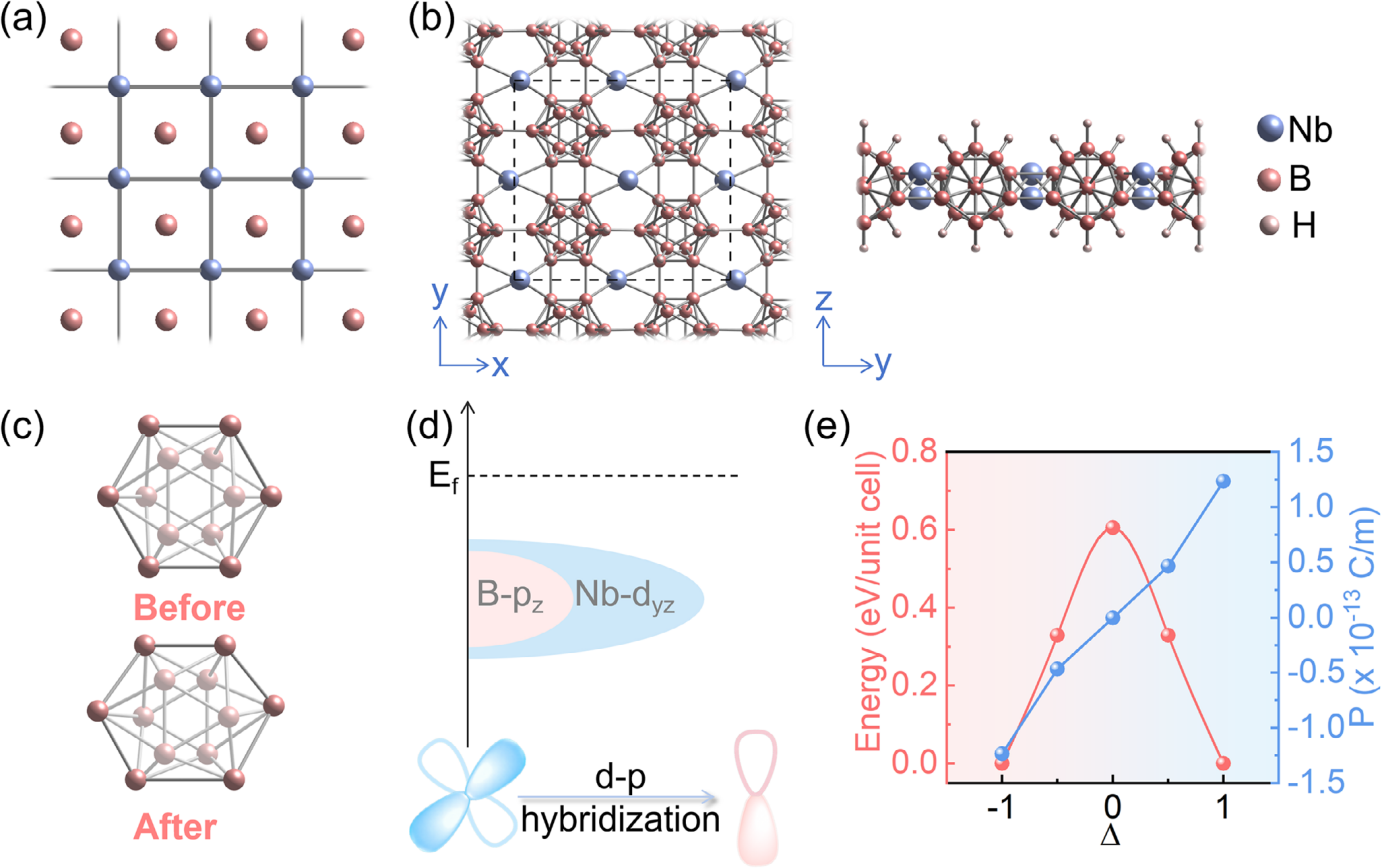
(a) A square lattice with fully occupied central sites. (b) Top and side views of NbB_12_H_6_ monolayer. (c) Structural deformation of the B_12_ cage before and after its incorporation into the Nb square lattice. (d) *d*‐*p* orbital hybridization between Nb and B atoms in NbB_12_H_6_ monolayer. (e) Ferroelectric polarization switching barrier and corresponding polarization magnitude in NbB_12_H_6_ monolayer. Δ is a dimensionless reaction coordinate describing the structural switching path (see Supporting Information for its definition).

To address the dynamic instability, we turn our attention to the 4*d* transition metal Nb. Possessing partially filled 4*d*‐orbitals, Nb facilitates stronger and more directional metal‐boron bonds with the *closo*‐dodecaborate superatom, thereby enhancing bond stiffness and lattice rigidity. Furthermore, increased charge transfer and orbital overlap between Nb and the boron cluster improve electronic screening and suppress soft phonon modes. Consistent with these considerations, embedding the dodecaborate superatom in an Nb‐based square lattice results in a dynamically stable monolayer with the chemical composition the NbB_12_H_6_ (Figure 2b; Figure S2a). Given the rotational degree of freedom of the superatomic building block, we further assessed the energetic stability of different orientational configurations within the 2D lattice. Total‐energy calculations for a set of rotated structures show that the orientation used in this work is the energetic minimum (Figure S3), while alternative configurations are consistently higher in energy. This demonstrates that the reported geometry is the preferred stable configuration. Structural analysis reveals substantial in‐plane and out‐of‐plane displacements of Nb atoms (Figure 2b), reflecting strong orbital coupling between the superatom and the metal framework. By contrast, Nb coordinated with conventional main‐group ligands retains a high‐symmetry square lattice (Figure 2a), underscoring the unique role of superatoms in enabling symmetry breaking required for ferroic ordering.

### 2.3 Origin of Structural Distortion

The pronounced displacement of Nb atoms is directly coupled to the distortion of the *closo*‐dodecaborate superatom. In its isolated form, the dodecaborate cage adopts *I_h_* symmetry [25], characterized by nearly equivalent B─B bond lengths (l = ∼1.76 Å) and bond angles (θ = ∼108°). When embedded in the 2D Nb square lattice, however, the local geometry of the B_12_ cage is strongly perturbed (Figure 2c). As shown in Figure S4, the B─B bond lengths and bond angles span a broad range from 1.75 to 1.89 Å and from 102.56° to 108.83°, respectively, indicating substantial symmetry lowering of the cage. Bader charge analysis reveals pronounced charge inhomogeneity among the peripheral B atoms, with charge variations ranging from 0.09 to 0.46 e^−^ (Table S1). This uneven charge distribution directly disrupts the equivalence of the B─B bonding network and provides a microscopic origin for the cage distortion. The projected density of states (PDOS) analysis (Figure S5) shows that electronic states near the Fermi level are dominated by Nb *d*‐orbitals and B *p*‐orbitals from the superatom. In particular, within the energy window from −2 to 0 eV, these states exhibit strong overlap, with the Nb *d_yz_*‐ and B *p_z_*‐orbitals displaying nearly complete hybridization (Figure 2d), evidencing strong metal‐superatom coupling.

**FIGURE 3:**
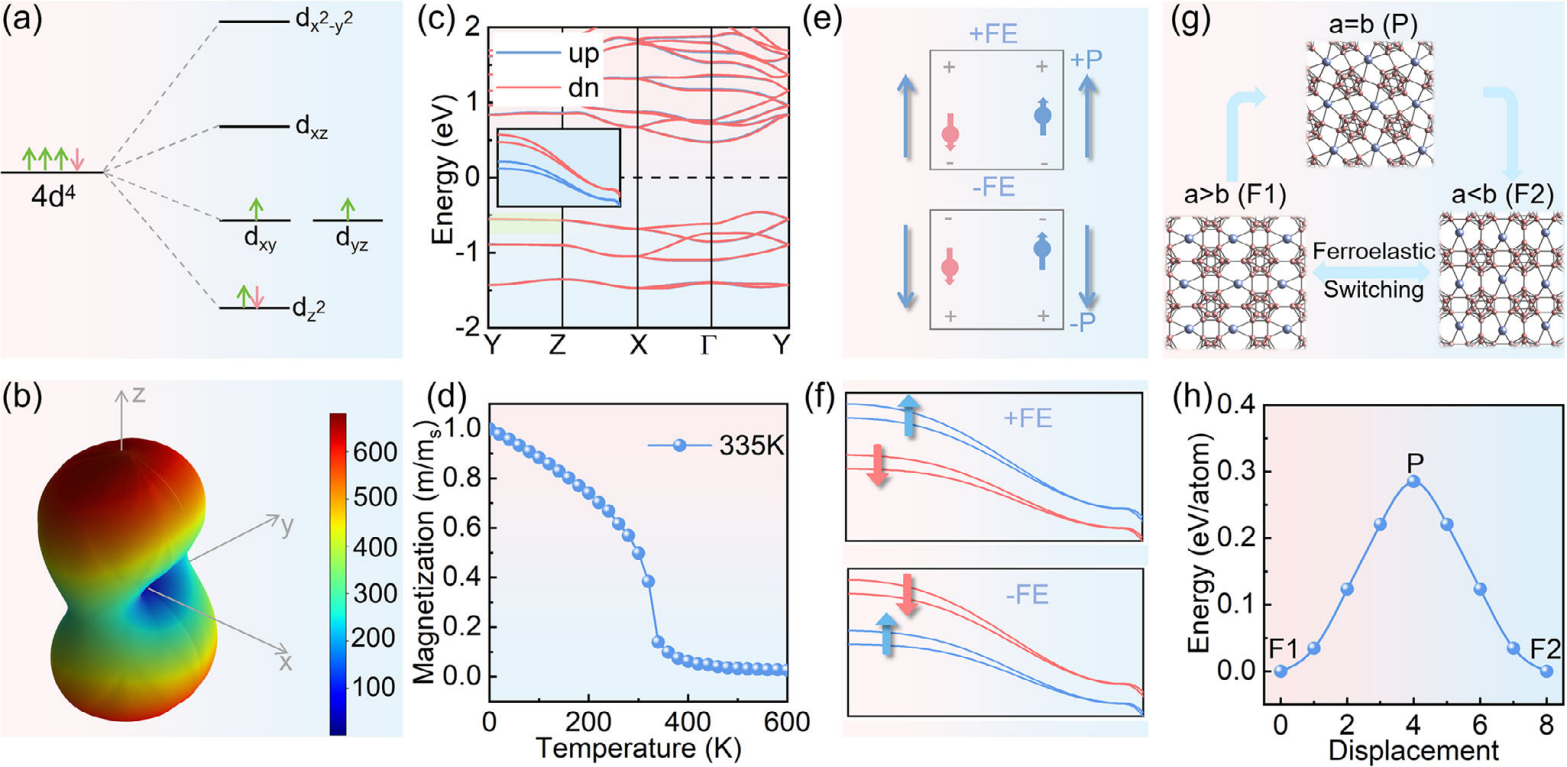
(a) Orbital‐level splitting of Nb atoms in NbB_12_H_6_ monolayer. (b) Angular dependence of MAE with the magnetization oriented in three‐dimensional space. (c) Electronic band structure of NbB_12_H_6_, highlighting the spin splitting along the Y‐Z direction. (d) Temperature dependence of the normalized magnetic moment of NbB_12_H_6_. (e,f) Schematic illustration of ferroelectrically controlled spin reversal in the AFM ground state and the corresponding spin‐reversal response in the band structure along the Y‐Z direction. (g) FA switching among three variants: the initial FA state (F1), the paraelastic state (P), and the final FA state (F2). (h) Energy profile of the FA transition from the F1 state to the F2 state in NbB_12_H_6_ monolayer.

Further charge analysis indicates that each Nb atom loses approximately one electron upon coordination with the superatom, leading to an effective valence change from 4*d* ^4^5*s* ^1^ to 4*d* ^4^. In a square crystal‐field environment, a *d* ^4^ electronic configuration is intrinsically susceptible to Jahn‐Teller distortion. Here, the strong *d‐p* hybridization between Nb and the B_12_ cage introduces an electronic instability that drives a reorganization of the Nb *d*‐orbital energy levels. Orbital‐resolved analysis reveals that the degeneracy between the *d_xz_*‐ and *d_yz_*‐orbitals in the ideal square lattice is lifted, while the $d_{z^{2}}$‐orbital becomes the lowest‐energy state in the NbB_12_H_6_ monolayer (Figure 3a). This orbital reordering leads to a redistribution of electronic charge, which in turn induces lattice relaxation, lowers the total energy, and breaks the original symmetry, ultimately stabilizing a polar structure.

### 2.4 Ferroelectricity

In the NbB_12_H_6_ monolayer, displacements of Nb ions along the x and z directions generate both in‐plane and out‐of‐plane electric polarizations (Figure S6). Our calculations yield a spontaneous in‐plane polarization of 3.06 × 10^−14^ C/m, while the out‐of‐plane component reaches 1.24 × 10^−13^ C/m, which is approximately one order of magnitude larger [26]. As shown by the light‐blue curve in Figure 2e, the out‐of‐plane polarization is comparable to that reported for MoSi_2_N_4_ (1.8 × 10^−13^ C/m) [27] and InSe (2.4× 10^−13^ C/m) [28], and notably exceeds that of the ReS_2_ monolayer (0.7 × 10^−13^ C/m) [29].

### 2.5 Antiferromagnetism and Spin‐Splitting Band Structure

The sizable ferroelectric polarization in the NbB_12_H_6_ monolayer motivates further investigation of its magnetic properties. We first verify that the NbB_12_H_6_ monolayer is dynamically, thermally, and mechanically stable (Figure S2 and Table S2). A comparison of different magnetic configurations identifies the AFM3 configuration as the magnetic ground state (Figure S8). The magnetic moments are primarily localized on the Nb atoms, each carrying a moment of approximately 1.83 *µ_B_*. To elucidate the microscopic origin of magnetism, we analyze the Nb *d*‐orbital splitting, as illustrated in Figure 3a. The five *d*‐orbitals split into four energy levels, with the $d_{x^{2}-y^{2}}$‐orbital at the highest energy and the $d_{z^{2}}$‐orbital at the lowest. As discussed above, the Nb valence configuration is 4*d^4^*, with electrons occupying the lower‐energy $d_{z^{2}}$ and *d_xy_*/*d_yz_*‐orbitals. The presence of unpaired electrons in these orbitals gives rise to a local magnetic moment of approximately 2 *µ_B_* per Nb atom, in good agreement with the calculated value. Notice that, for the designed structure, the host lattice need not be intrinsically magnetic. In principle, superatom‐induced charge transfer and ligand‐field modification may also generate or stabilize magnetism. In the present NbB_12_H_6_ system, however, we found that the Nb host lattice without superatom already carries local moments, and the role of the superatom is to modulate these moments and induce the symmetry‐lowering distortion required for ferroicity.

The magnetic anisotropy energy (MAE) of the NbB_12_H_6_ monolayer is presented in Figure 3b. When the spins are aligned perpendicular to the plane (θ = 0° or 180°), the MAE reaches a maximum value of 680 µeV per Nb atom. This value exceeds those reported for many previously studied 2D magnetic systems, including Cr_2_Ge_2_Te_6_ (660 µeV/Cr) [30], NiPS_3_ (82.7 µeV/Ni) [31], MnN (400 µeV/Mn) [32], Ca(pyz)_2_ (0.21 µeV/u.c) and Sr(pyz)_2_ (0.95 µeV/u.c) [33]. We further estimate the Néel temperature T_N_ using Monte Carlo (MC) simulations (see Supporting Information for details) [34]. By incorporating both nearest‐ and next‐nearest‐neighbor exchange interactions (*J*_1_ and *J*_2_), the calculated T_N_ reaches 335 K, well above room temperature (Figure 3d). This value significantly exceeds those of experimentally realized 2D magnets, such as CrI_3_ (∼45 K), Cr_2_Ge_2_Te_6_ (∼80 K), and Fe_3_GeTe_2_ (∼130 K) [35, 36, 37]. The combination of large MAE and high T_N_ indicates the exceptional magnetic robustness of the NbB_12_H_6_ monolayer, highlighting its potential for applications in 2D spintronics and information‐storage devices.

Figure 3c shows the electronic band structure of the NbB_12_H_6_ monolayer without considering spin‐orbit coupling (SOC). The system is an indirect semiconductor with a band gap of 0.93 eV. Notably, despite its AFM ground state with zero net magnetic moment, pronounced spin splitting emerges along all high‐symmetry paths when the band dispersions are examined in detail. The maximum spin‐splitting energy reaches 13.36 meV along the X‐Γ path. Because this band splitting is not momentum‐locked, the system is not classified as AMs. Instead, the NbB_12_H_6_ monolayer belongs to the class of fully compensated AFM exhibiting spin‐splitting band structures (AFM‐ss). This can be further explained through symmetry analysis. In the undistorted, ideal NbB_12_H_6_ lattice, the magnetic space group is *C*2*m*′(No. 12.61), which preserves the combined *PT* symmetry. Upon full structural relaxation, however, both the superatomic units and the Nb square lattice experience symmetry lowering, leading to the breaking of *PT* symmetry. Consequently, the AFM spin‐split band structure emerges in the relaxed AFM phase.

Importantly, reversing the ferroelectric polarization from the upward (↑) to the downward (↓) direction leads to a complete reversal of the spin splitting in the electronic band structure (Figure 3e,f; Figure S9). This behavior demonstrates that the AFM‐ss can be directly controlled via FE polarization switching. Previous studies have reported ferroelectrically switchable spin splitting in a limited number of alter‐ and antiferromagnetic systems, typically arising from intrinsic structural asymmetry [13, 14, 15, 38, 39]. However, a general and designable strategy to achieve ferroelectrically tunable spin splitting in conventional materials has remained elusive. By embedding superatoms into an otherwise centrosymmetric lattice, the present approach introduces controllable structural distortion that simultaneously induces FE and spin splitting, providing a viable route toward electrically tunable spin textures in 2D AFMs.

We further examine the influence of SOC on the electronic structure. Upon including SOC, the band splitting along the Y‐Z high‐symmetry path becomes more pronounced, and the band gap slightly increases to 0.95 eV (Figure S10a). To gain deeper insight into the SOC‐induced spin textures, we analyze the spin distribution near the Γ point for both the valence band maximum (VBM) and the conduction band minimum (CBM) (Figure S10b,c). In both cases, the spin polarization is dominated by out‐of‐plane components (S_z_ < 0), while their momentum‐dependent textures are markedly different. The VBM exhibits a localized enhancement of S_z_ along the k_x_ direction and a more uniform distribution along k_y_ direction, whereas the CBM shows the opposite behavior, with enhanced localization along k_y_ direction. This pronounced anisotropy in momentum space further confirms the presence of robust spin‐splitting features in the NbB_12_H_6_ monolayer.

### 2.6 Ferroelasticity

Ferroelastic (FA) materials are characterized by the existence of two or more energetically equivalent orientation variants, between which reversible switching can be achieved exclusively by applying external strain. As shown in Figure 3g, two energetically degenerate NbB_12_H_6_ monolayers are presented, labeled as the initial state (F1, a > b) and the final state (F2, a < b). An intermediate paraelastic state (P) exists between F1 and F2, where the lattice constants satisfy a = b. A key parameter governing FA performance is the reversible strain, defined as [(b/a – 1) × 100%]. This strain is directly related to the signal strength of FA switching. The calculated reversible strain for the NbB_12_H_6_ monolayer is 15.1%, which is comparable to that of *t*‐YN (14.4%) [40] and GeS (17.8%) [41], but significantly larger than those of SnS (4.9%), SnSe (2.1%) [41], and MoSSe (4.7%) [42]. The FA switching pathway is further examined using the nudged elastic band (NEB) method [43]. As shown in Figure 3h, the P state acts as a bridge connecting the F1 and F2 states, with no additional metastable states observed during the switching process. The calculated activation energy barrier for FA switching is 0.29 eV per atom, which is lower than that of BP_5_ (0.32 eV/atom) [44], LuSCl (0.30 eV/atom) [45], and ZrAsCl (0.33 eV/atom) [46]. In addition, we investigated the evolution of the magnetic ground state of NbB_12_H_6_ during the FA switching process (Figure S11). The results show that NbB_12_H_6_ remains in the AFM ground state throughout the entire switching pathway, with no change in its magnetic ordering. This relatively low activation barrier underscores the high efficiency of FA switching in the NbB_12_H_6_ monolayer.

Experimentally, the NbB_12_H_6_ layer can be assembled from the *closo*‐dodecaborate precursors. A feasible route is to deposit or react dodecaborate clusters on an Nb‐containing template, where partial removal of H atoms enables formation of Nb─B bonds while retaining the essential B_12_ cage framework. Under suitable surface‐confined conditions, such directed coupling could promote periodic occupation of equivalent lattice sites and thereby realize the designed architecture.

### 2.7 Materials Screening with Alternative Superatoms and Metal Atoms

We have established that a superatom can drive intrinsic triferroicity in a 2D square lattice. Importantly, the choice of the B_12_H_12_ superatom is not unique. By embedding an alternative superatom—such as experimentally accessible carborane—into the lattice, multiferroic behaviors can likewise be realized. The designed carborane‐based monolayer exhibits high thermodynamic and dynamic stability (Figures S12 and S13), an AFM ground state, a spontaneous polarization of 5.36 × 10^−15^ C/m, and a FE switching barrier of 2.89 eV per unit cell, indicative of a robust ferroelectric response. These results suggest that the broad family of superatoms provides substantial opportunities for tailoring and modulating ferroic properties.

**FIGURE 4:**
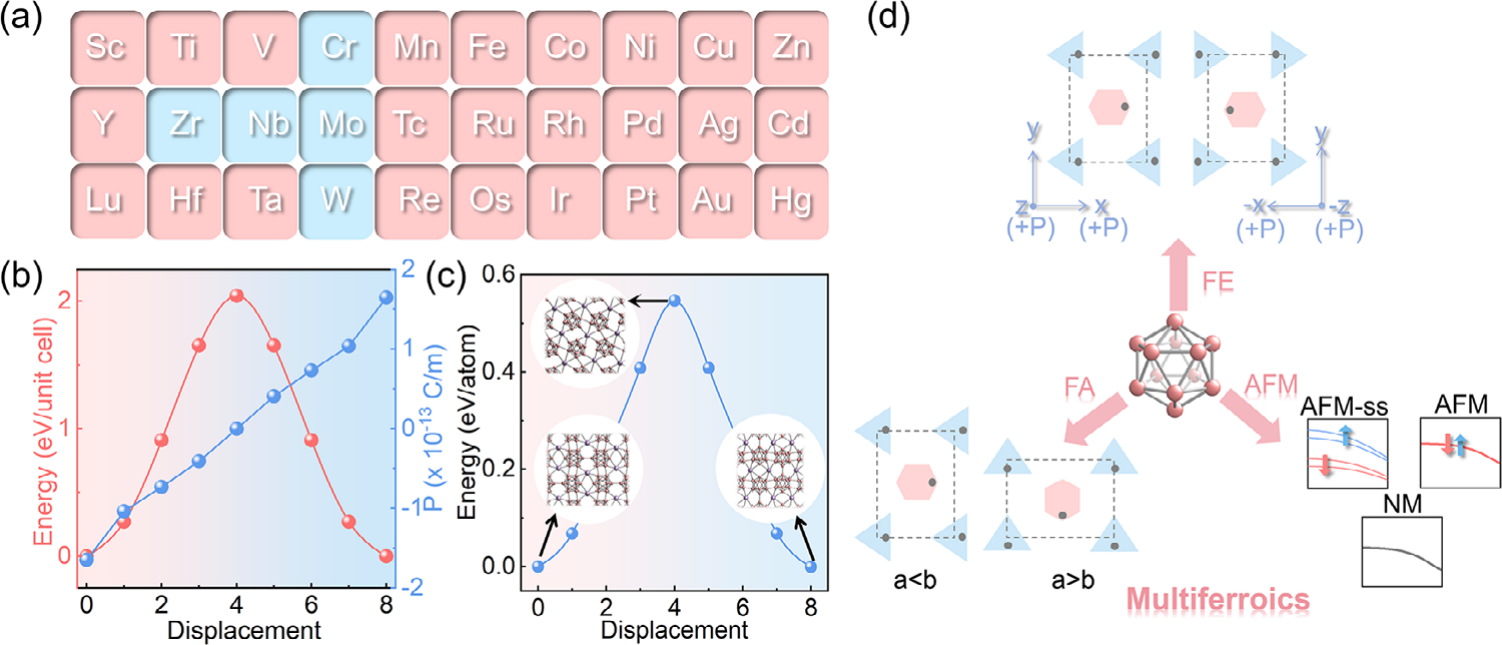
(a) Transition metals selected for materials screening, where pink denotes elements that do not show FE, and light blue denotes elements that exhibit FE. (b) FE switching pathways and associated energy barriers for the ZrB_12_H_6_ monolayer, yielding a spontaneous polarization of 1.65 × 10^−13^ C/m and a switching barrier of 2.04 eV per unit cell. (c) FA switching pathways and corresponding energy barriers for the ZrB_12_H_6_ monolayer, with a FA barrier of 0.55 eV per atom. (d) Conceptual illustration of superatom‐induced triferroic orders.

We further examine whether the Nb atom is uniquely responsible for inducing multiferroicity within this superatom‐based framework. To this end, Nb was systematically replaced by other transition‐metal elements (Figure 4a). Among the metals investigated, Cr‐, Mo‐, Zr‐, and W‐based 2D systems are identified as multiferroic. In the Cr‐ and Mo‐based lattices, AFM and FA orders coexist, whereas the W‐based monolayer exhibits coupled FE and FA orders. Notably, ZrB_12_H_6_ simultaneously hosts FE (Figure 4b), AFM, and FA (Figure 4c) orders, rendering it another promising triferroic candidate (Figure 4d). Detailed analyses of structural stability, magnetic configurations, and FE and FA switching pathways are provided in Figures S14–S19. These results demonstrate that the diversity in both superatomic motifs and transition‐metal element selection provides expanded opportunities for the future design of novel superatom‐based 2D multiferroic materials.

## 3 Conclusion

In this work, we have developed a general strategy for realizing 2D triferroics that simultaneously host FE, FA, and AFM‐ss through the incorporation of superatoms. By leveraging strong *p*‐*d* orbital hybridization between superatoms and transition‐metal atoms, we demonstrate that superatom‐induced symmetry‐lowering distortions can effectively break inversion symmetry and stabilize coupled ferroic orders within a single lattice. Using the NbB_12_H_6_ monolayer as a representative system, we show that the Jahn‐Teller effect plays a key role in driving structural distortion, which in turn enables the emergence of FE polarization, robust AFM with sizable spin splitting, and FA switching with a low activation barrier. Notably, the spin‐splitting state can be deterministically reversed via FE switching, highlighting strong magnetoelectric coupling and offering a practical route toward electrically controllable spin functionalities.

Beyond the prediction of a specific material, this work establishes a previously unexplored design principle for 2D triferroics with AFM‐ss. The central conceptual advance lies in introducing superatoms as symmetry‐active building blocks: unlike conventional atoms, superatoms possess internal structural flexibility and multicenter bonding, enabling cooperative symmetry breaking that is inaccessible in ordinary atomic lattices. Within this framework, we demonstrate that superatom incorporation can simultaneously generate FE, FA, and AFM spin splitting in a single monolayer, while enabling deterministic electrical control of the spin‐splitting state via ferroelectric switching. Importantly, this mechanism is not limited to a specific material but provides a general and transferable strategy for designing multifunctional 2D systems through superatom engineering.

## 4 Computational Method

The structural relaxations were performed on the basis of density functional theory (DFT) as implemented in the Vienna ab initio Simulation Package (VASP) [47]. The electronic exchange‐correlation functional was treated by the generalized gradient approximation (GGA) proposed by Perdew, Burke, and Ernzerhof (PBE) [48]. To deal with the strongly correlated 3*d*‐ and 4*d*‐orbitals of Cr, Mo, Zr, and Nb, the Hubbard correction was used with the value U of 4.0 eV [49]. To consider the weak van der Waals (vdW) interactions between interlayers, we used the Grimme DFT‐D3 approach [50]. The energy cutoff of the plane waves was set to 500 eV. The structures were fully relaxed until the maximum force on each atom was less than 0.005 eV/Å. The energy convergence criterion in the self‐consistent calculations was set to 10^−6^ eV. A Gamma‐centered Monkhorst‐Pack *k*‐point mesh with a resolution of 2π × 0.03 Å^−1^ was used for geometry optimization and self‐consistent calculations. A vacuum slab of at least 20 Å in *z* direction was adopted to avoid artificial interactions between the neighboring layers. The phonon dispersion was computed by using the Phonopy code [51] within the density functional perturbation theory (DFPT) [52]. In phonon calculations, a finer *k*‐point grid of 2π × 0.02 Å^−1^ was employed. Ab initio molecular dynamics (AIMD) simulations were performed at 500 K within 10 ps to evaluate the thermal stability of the NbB_12_H_6_ monolayer. The ferroelectric polarization was calculated using the Berry‐phase formalism [53] combined with the dipole correction method [54].

The simulations were based on the classical Heisenberg spin Hamiltonian:

(1) $$H\;=-\sum_{i<j}J_{ij}S_{i}S_{j}-k\sum_{i}{\left(S_{i}^{z}\right)}^{2}$$

where **S**_*i*_ and **S**_*j*_ are unit vectors describing the spin orientations at sites *i* and *j*, respectively. *k*is the magnetic anisotropy constant, and $S_{i}^{z}$ is the out‐of‐plane component of the spin. For the NbB_12_H_6_ monolayer, the MC simulations were performed on a simulation cell of 30 nm × 30 nm, containing 3600 Nb atoms. The spins were initialized along the easy axis and equilibrated for 2 × 10^4^ MC steps, followed by 5 × 10^4^ additional steps for thermal averaging at each temperature. The Hinzke–Nowak combinational algorithm [55] was employed for efficient relaxation toward thermal equilibrium, and periodic boundary conditions were applied throughout.

The nearest‐neighbor (*J*_1_) and next‐nearest‐neighbor (*J*_2_) exchange parameters were extracted using the following energy relations:

(2) $$E_{\mathrm{FM}}=E_{0}\;-4J_{1}|S{|}^{2}-4J_{2}|S{|}^{2}$$

(3) $$E_{\mathrm{AFM}2}=E_{0}+4J_{1}|S{|}^{2}-4J_{2}|S{|}^{2}$$

(4) $$E_{\mathrm{AFM}3}=E_{0}+4J_{1}|S{|}^{2}+4J_{2}|S{|}^{2}$$

where *E*_0_ is the reference magnetic energy, and *E*_FM_,*E*_AFM2_,*E*_AFM3_ denote the DFT total energies of the corresponding magnetic configurations.

## Conflicts of Interest

The authors declare no conflicts of interest.

## Supporting information




**Supporting File**: advs75168‐sup‐0001‐SuppMat.docx.

## Data Availability

The data that support the findings of this study are available from the corresponding author upon reasonable request.
