# Supplementary material for: Superatom Distortion Induces Triferroicity and Spin Splitting in Two‐Dimensional Antiferromagnets
Source: Adv Sci (Weinh). 2026 Apr 7:e00005. Online ahead of print. doi: 10.1002/advs.202600005 (PMC13325812; doi:10.1002/advs.202600005)
Supplement: Supplementary file 1 — Supporting File: advs75168‐sup‐0001‐SuppMat.docx. [file ADVS-9999-e00005-s001.docx]

**Supporting Information**

**Superatom Distortion Induces Triferroicity and Spin Splitting in Two-Dimensional Antiferromagnets**

*Zhen Gao, Fengxian Ma, Guoping Gao,^*^ Weizhen Meng, ^*^ Ying Liu, Yandong Ma,^*^ and Yalong Jiao^*^*

*Zhen Gao, Fengxian Ma, Weizhen Meng, Ying Liu, Yalong Jiao*

*College of Physics, Hebei Key Laboratory of Photophysics Research and Application, Hebei Normal University, Shijiazhuang 050024, China.*

*E-mail:* [*mengweizhen@hebtu.edu.cn*](mailto:mengweizhen@hebtu.edu.cn)*;* [*yalong.jiao@hebtu.edu.cn*](mailto:yalong.jiao@hebtu.edu.cn)

*Guoping Gao*

*MOE Key Laboratory for Non-equilibrium Synthesis and Modulation of Condensed Matter, Shaanxi Province Key Laboratory of Advanced Functional Materials and Mesoscopic Physics, School of Physics, Xi’an Jiaotong University, Xi’an, Shaanxi 710049, China.*

*E-mail:* [*guopinggao@xjtu.edu.cn*](mailto:guopinggao@xjtu.edu.cn)

*Yandong Ma*

*School of Physics, State Key Laboratory of Crystal Materials, Shandong University, Jinan 250100, China.*

*E-mail:* [*yandong.ma@sdu.edu.cn*](mailto:yandong.ma@sdu.edu.cn)

Table of Contents

[1. Calculation of Dimensionless Parameter in Ferroelectric Switching S3](#_Toc224758472)

[2. Magnetic Ground State and Stability S3](#_Toc224758473)

[3. Magnetocrystalline Anisotropy Energy S4](#_Toc224758474)

[4. Crystal structure and phonon dispersions of the checkerboard-lattice Cu_2_B_12_H_6_ monolayer S5](#_Toc224758475)

[5. Phonon dispersion and AIMD simulation of NbB_12_H_6_ monolayer S5](#_Toc224758476)

[6. Energy Profile of NbB_12_H_6_ monolayer at Superatom Rotation Angles S6](#_Toc224758477)

[7. Bond lengths in the distorted B_12_H_6_ cluster S6](#_Toc224758478)

[8. PDOS for the NbB_12_H_6_ monolayer S6](#_Toc224758479)

[9. Schematic of the Ferroelectric sliding S7](#_Toc224758480)

[10. Magnetic Configurations of NbB_12_H_6_ Monolayer S7](#_Toc224758481)

[11. Band structure modulation via atomic displacement in the –FE state of NbB_12_H_6_ S8](#_Toc224758482)

[12. Band structure and spin texture of NbB_12_H_6_ monolayer with the SOC S8](#_Toc224758483)

[13. Ferroelastic Transition Energy Profile of NbB_12_H_6_ in NEB Calculations S9](#_Toc224758484)

[14. Materials Screening with Alternative Superatoms and Metal Atoms S9](#_Toc224758485)

[15. Bader charge analysis of the distorted boron cage S13](#_Toc224758486)

[16. The elastic constants of NbB_12_H_6_ monolayer S13](#_Toc224758487)

# **Calculation of Dimensionless Parameter in Ferroelectric Switching**

As shown in Figure S7, we introduce a dimensionless parameter Δ to characterize the reaction coordinate, defined as

$\Delta=\frac{\delta-\gamma}{\delta_{0}-\gamma_{0}}$

Here, δ₀ and γ₀ denote the height difference along the *z*-direction between Nb atoms (see Figure S7) in the ferroelectric ground state, while δ and γ correspond to the same quantities evaluated for intermediate configurations along the switching pathway. Under this definition, Δ varies continuously from −1 to 1, corresponding to the reversal of polarization from the negative to the positive orientation (see Figure 2e).

# **Magnetic Ground State and Stability**

To determine the magnetic ground state of the NbB_12_H_6_, one ferromagnetic (FM) and three antiferromagnetic (AFM) configurations were constructed (Figure S6). The AFM3 configuration exhibits the lowest total energy, indicating that it is the magnetic ground state. The magnetism originates primarily from the Nb atoms, each carrying a magnetic moment of approximately 1.83 *μ_B_*, close to 2 *μ_B_*. Then, we assessed the structural stability of the NbB_12_H_6_. The phonon spectrum shows no imaginary modes throughout the entire first Brillouin zone (Figure S2(a)), confirming its dynamical stability. Moreover, *ab initio* molecular dynamics (AIMD) simulations performed at 500 K for 10 ps reveal that the structure remains intact with only minor fluctuations in total energy (Figure S2(b)), demonstrating excellent thermal stability. To evaluate its mechanical stability, we computed the elastic constants (Table S2). The NbB_12_H_6_ monolayer satisfies the Born–Huang’s mechanical stability criteria ($C_{11}，C_{66}>0$ and $C_{11}C_{22}-C_{12}^{2}>0$) , confirming its mechanical stability ^[1]^. Overall, the outstanding dynamical, thermal, and mechanical stabilities of the NbB_12_H_6_ suggest that this material is potentially synthesizable in experiments.

# **Magnetocrystalline Anisotropy Energy**

Magnetocrystalline anisotropy energy (MAE) is a key physical quantity governing the magnetic stability of low-dimensional magnetic materials. In two-dimensional (2D) systems, thermal fluctuations are particularly pronounced, and long-range magnetic order can be sustained at finite temperatures only when the MAE is sufficiently large to suppress spontaneous spin reorientation. The MAE quantitatively characterizes the energy barrier that must be overcome when rotating the magnetization from the easy axis to the hard axis, and is commonly defined as

$$\mathrm{MAE}(\theta,\varphi)=E(\theta,\varphi)-E(\theta={90}^{\circ},\varphi=0^{\circ}),$$

where $\theta$ and $\varphi$ denote the polar and azimuthal angles describing the spin orientation relative to the crystal lattice.

The calculated MAE profile of the NbB_12_H_6_ monolayer is shown in Figure 3(b). Compared with in-plane orientations, spins aligned along the out-of-plane direction exhibit significantly lower energy, indicating that the magnetic easy axis lies perpendicular to the plane. When the spins are fully oriented out of plane ($\theta=0^{\circ}$ or ${180}^{\circ}$), the MAE reaches a maximum value of 680 μeV/Nb, which is substantially larger than many previously reported 2D magnetic systems, such as Cr_2_Ge_2_Te_6_ (660μeV/Cr) ^[2]^, NiPS_3_ (82.7μeV/Ni) ^[3]^, MnN(400μeV/Mn) ^[4]^, 2D Ca(pyz)_2_ (0.21μeV/u.c) and Sr(pyz)_2_ (0.95 μeV/u.c) ^[5]^. This remarkably large MAE not only ensures strong magnetic robustness of the NbB_12_H_6_ against thermal perturbations, but also highlights its promising potential for applications in spintronic and information-storage devices.

# **Crystal structure and phonon dispersions of the checkerboard-lattice Cu_2_B_12_H_6_ monolayer**


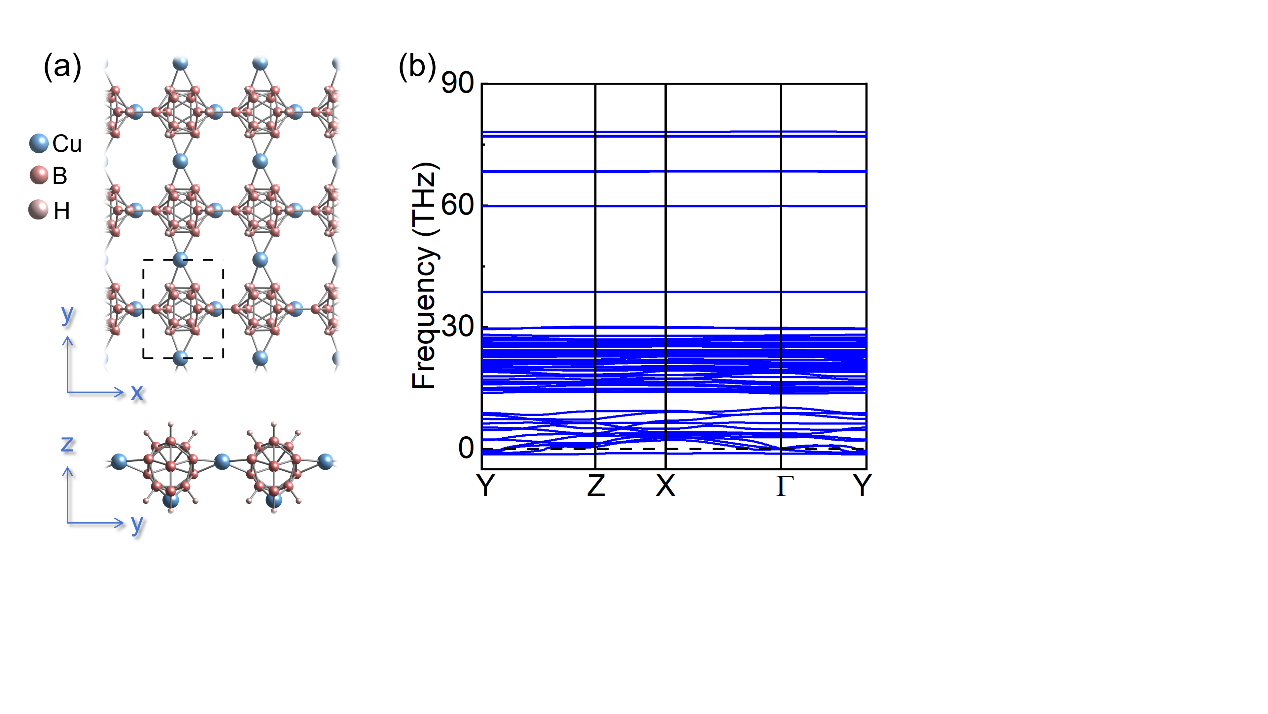


**Figure S1.** (a) Top and side views of Cu_2_B_12_H_6_ monolayer. (b) Phonon dispersions of Cu_2_B_12_H_6_ monolayer.

# **Phonon dispersion and AIMD simulation of NbB_12_H_6_ monolayer**


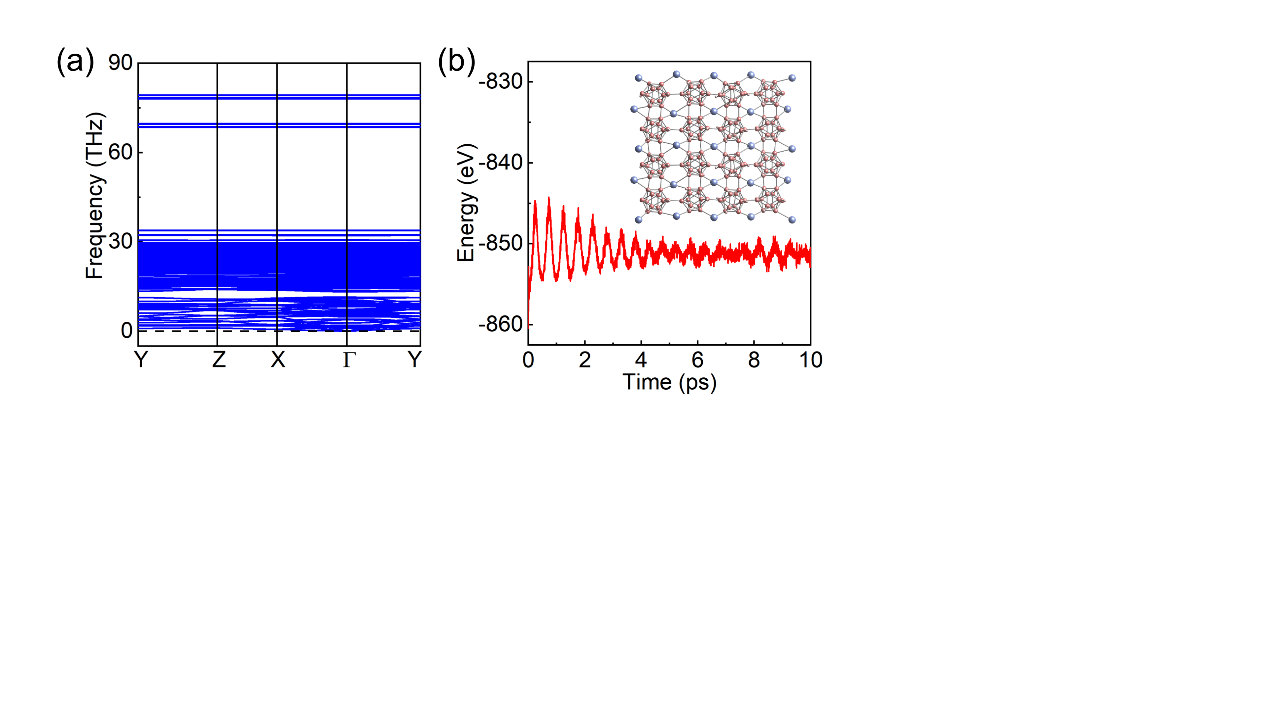


**Figure S2.** (a) Phonon dispersions and (b) total energy fluctuation during the AIMD simulation for NbB_12_H_6_. The inset of (b) is the structure of NbB_12_H_6_ after 10 ps.

# **Energy Profile of NbB_12_H_6_ monolayer at Superatom Rotation Angles**


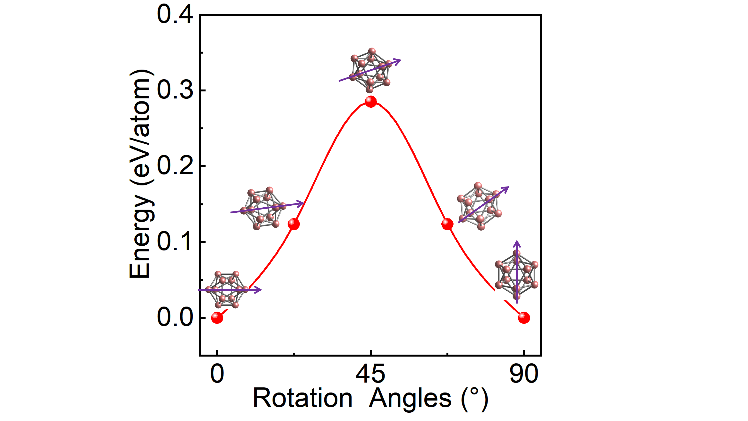


**Figure S3.** Energy profile of the NbB_12_H_6_ monolayer at varying rotational angles of the superatom, with respect to the x-y plane.

# **Bond lengths in the distorted B_12_H_6_ cluster**


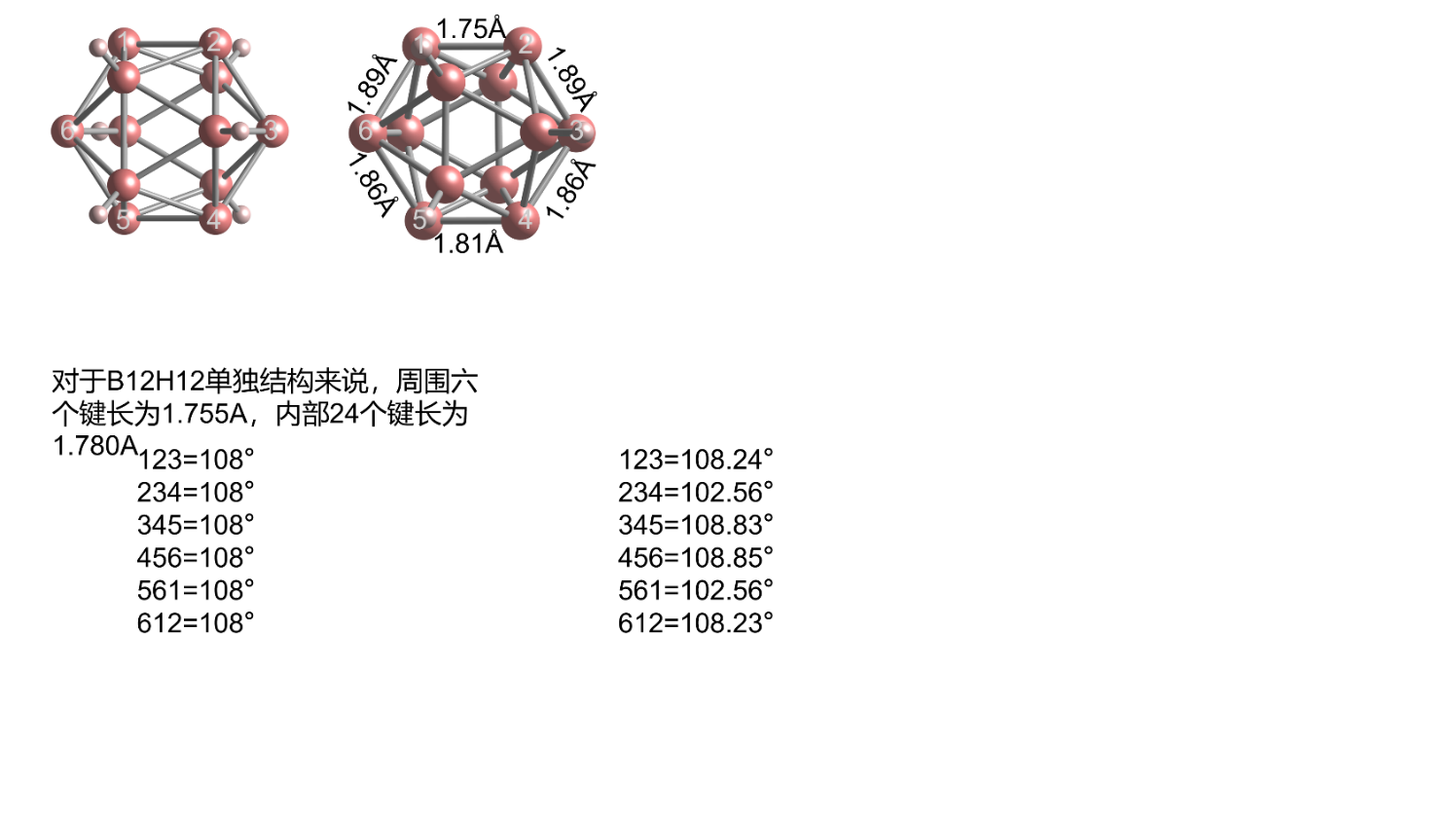


**Figure S4.** Band lengths of the *closo*-dodecaborate superatom in NbB_12_H_6_ monolayer.

# **PDOS for the NbB_12_H_6_ monolayer**


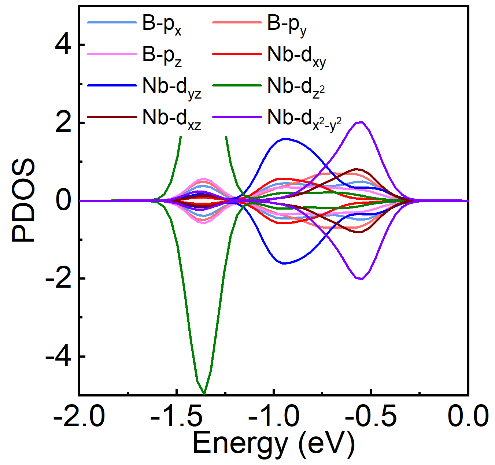


**Figure S5.** PDOS for the NbB_12_H_6_ monolayer.

# **Schematic of the Ferroelectric sliding**


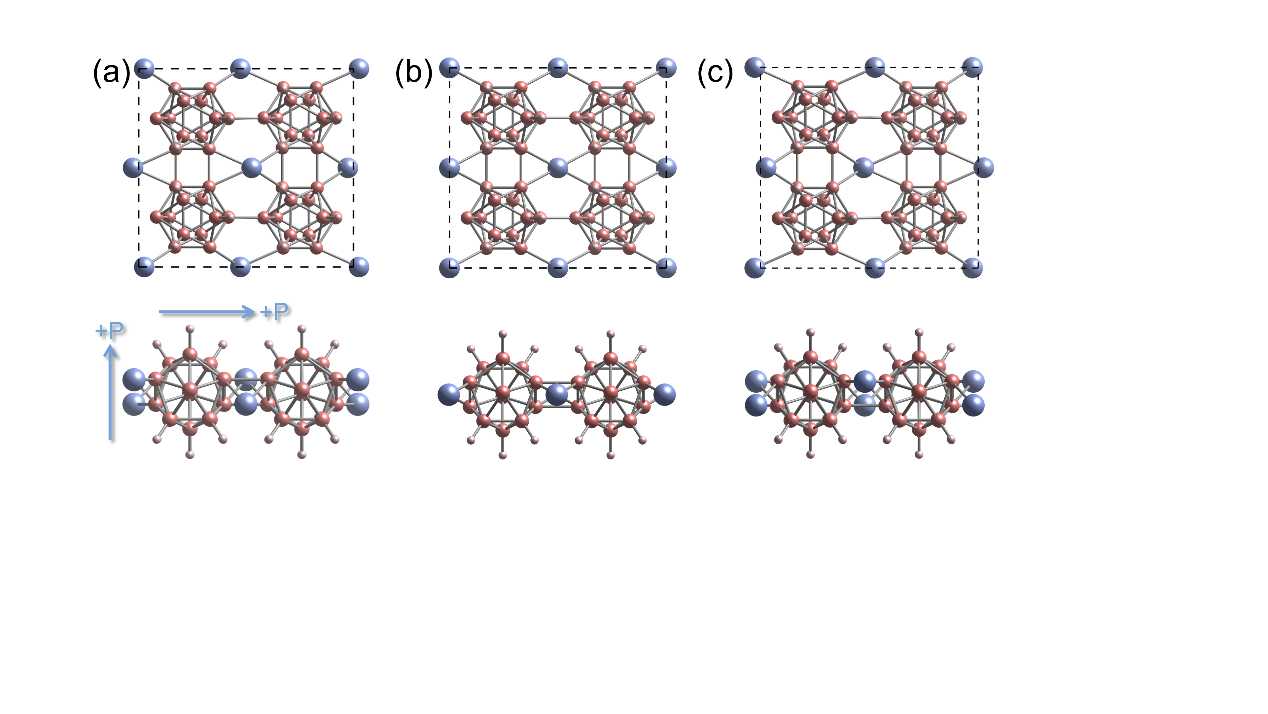


**Figure S6.** Schematic illustration of NbB_12_H_6_ monolayer after ferroelectric sliding, corresponding to the (a) +FE, (b) PE, and (c) –FE states.


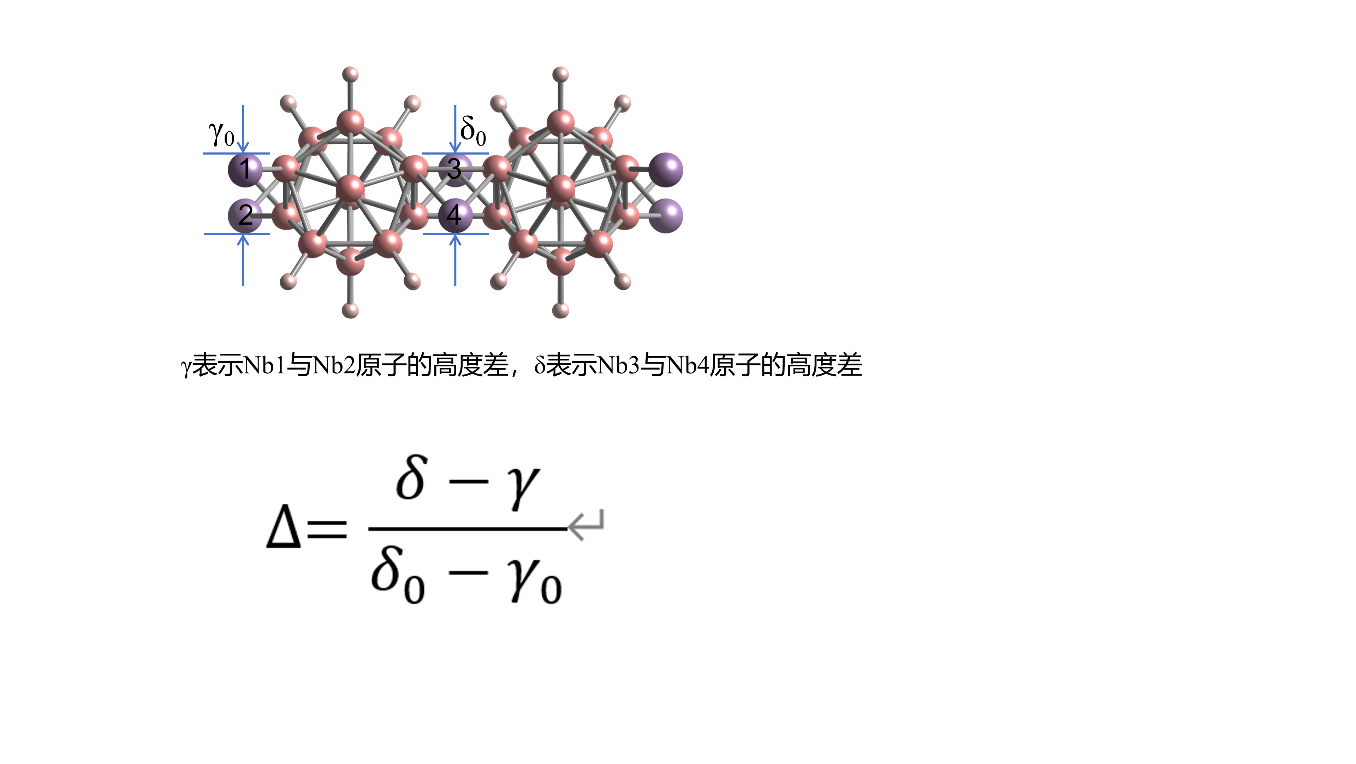


**Figure S7.** Side view of NbB_12_H_6_ monolayer, where $\delta_{0}$ and $\gamma_{0}$ represent the height differences between different Nb atoms.

# **Magnetic Configurations of NbB_12_H_6_ Monolayer**


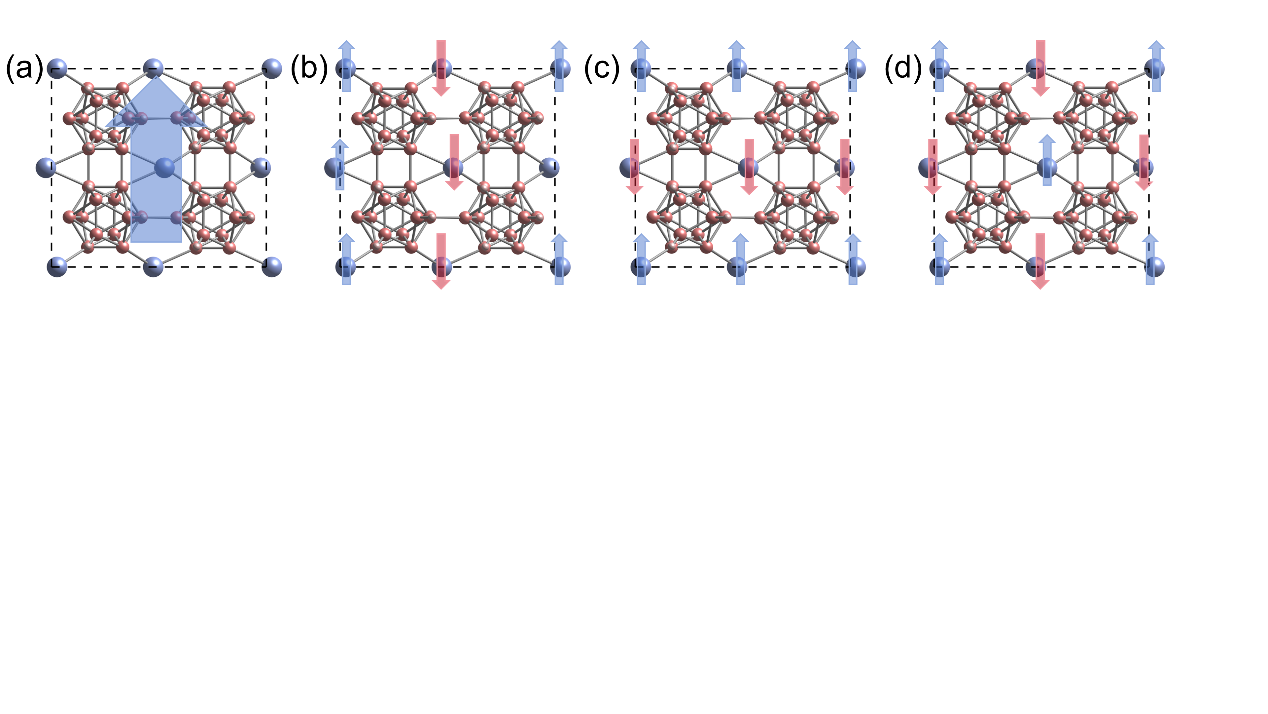


**Figure S8.** (a-d) FM and AFM configurations considered in our calculation. Red and blue arrows represent sites with opposite local spin directions.

# **Band structure modulation via atomic displacement in the –FE state of NbB_12_H_6_**


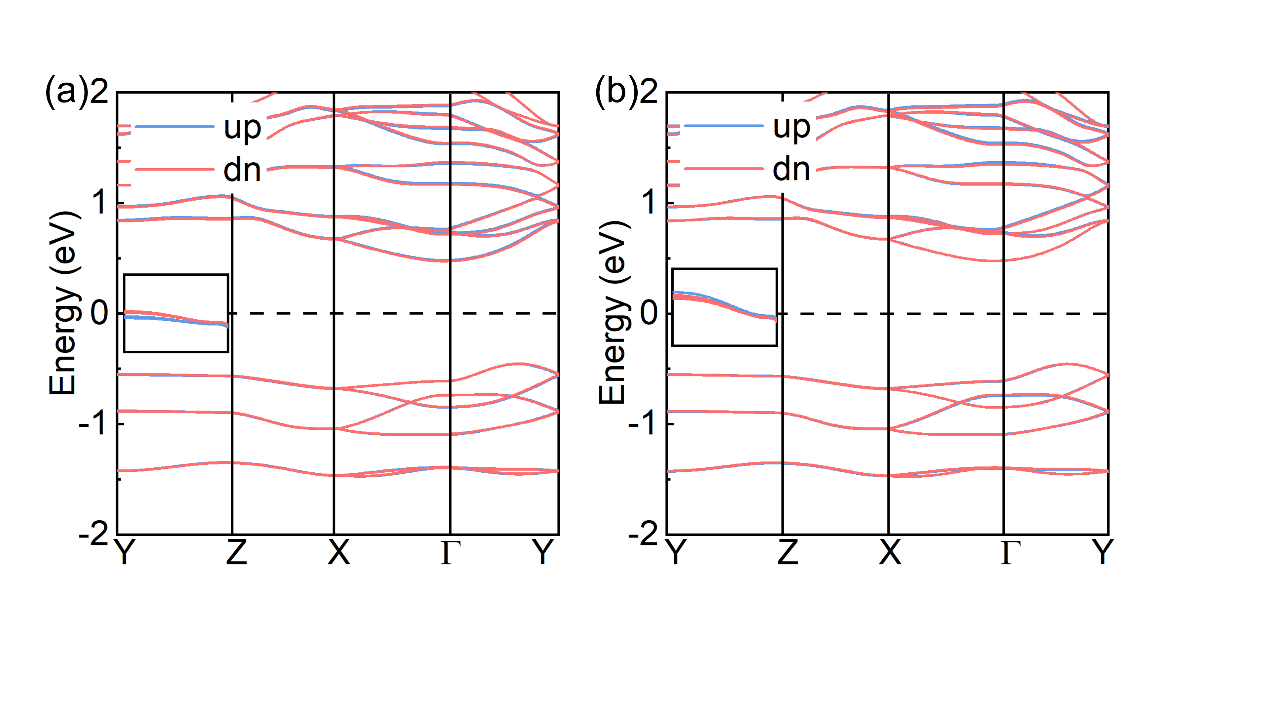


**Figure S9.** (a) Band structure of the −FE state after full structural relaxation. (b) Band structure of the −FE state obtained by manually shifting Nb atoms to enhance the ferroelectric distortion.

# **Band structure and spin texture of NbB_12_H_6_ monolayer with the SOC**


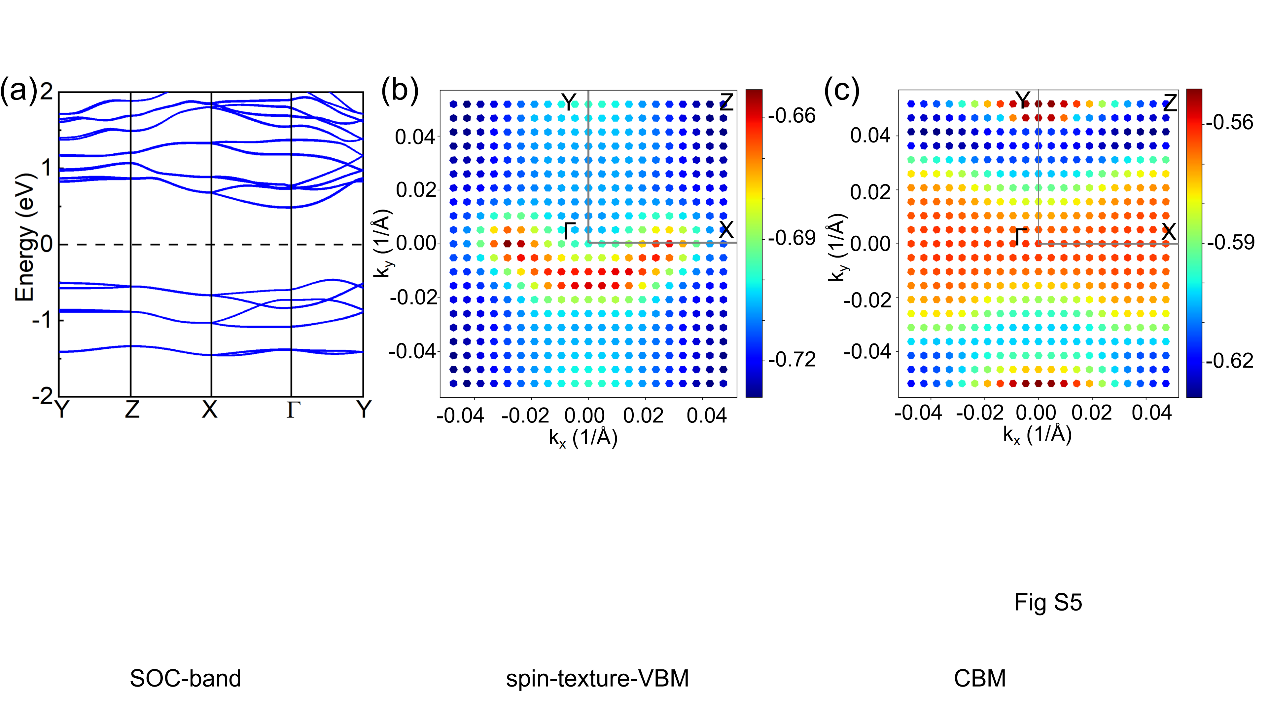


**Figure S10.** (a) Band structure of NbB_12_H_6_ with the SOC effect. (b-c) Spin textures for VBM and CBM of NbB_12_H_6_.

# **Ferroelastic Transition Energy Profile of NbB_12_H_6_ in NEB Calculations**


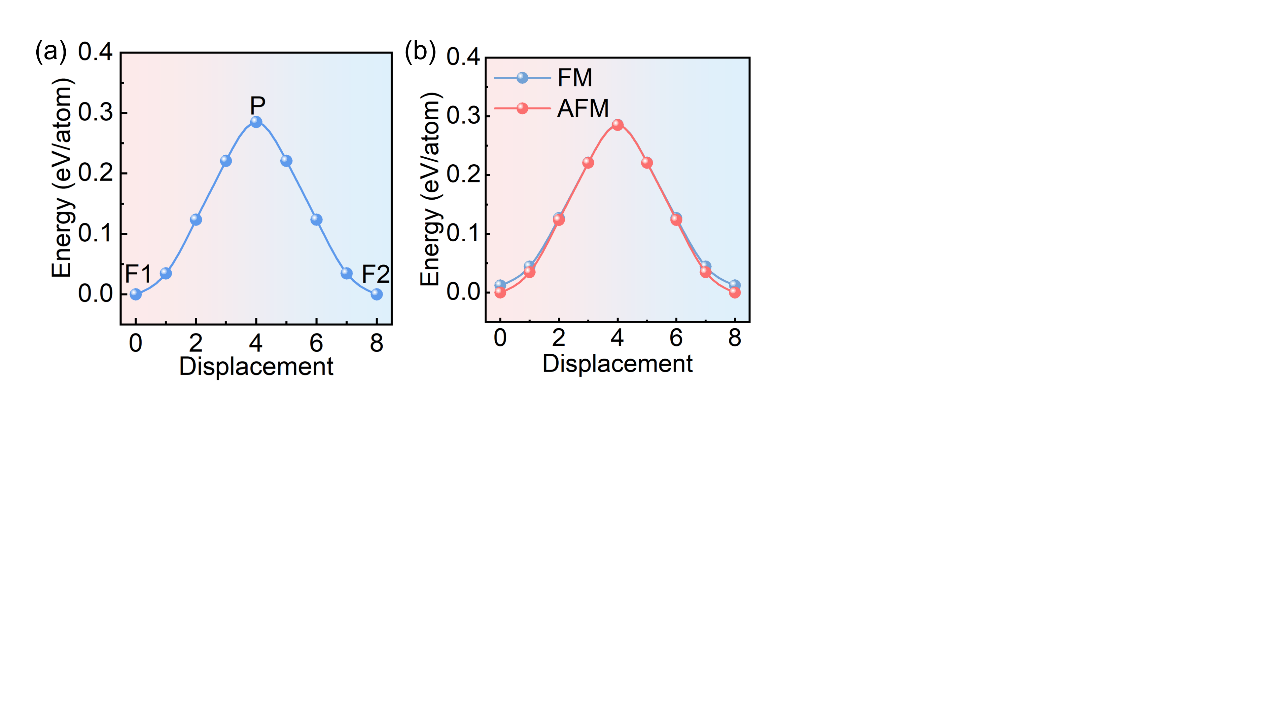


**Figure S11.** (a) Energy profile of the FA transition from the F1 state to the F2 state in NbB_12_H_6_ monolayer. (b) Energy comparison of NbB_12_H_6_ monolayer from the F1 state to the F2 states in different magnetic ground states.

# **Materials Screening with Alternative Superatoms and Metal Atoms**


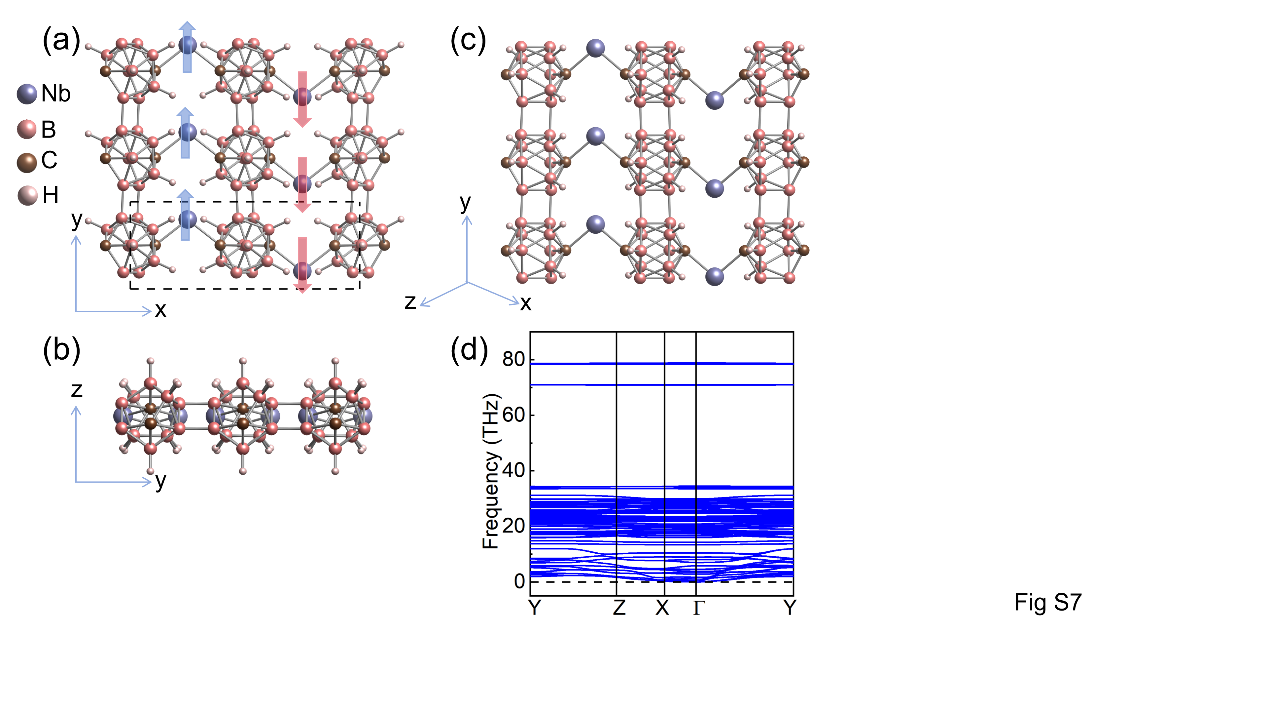


**Figure S12.** (a-b) Structure and (c) phonon spectrum of the NbB_10_C_2_H_6_.


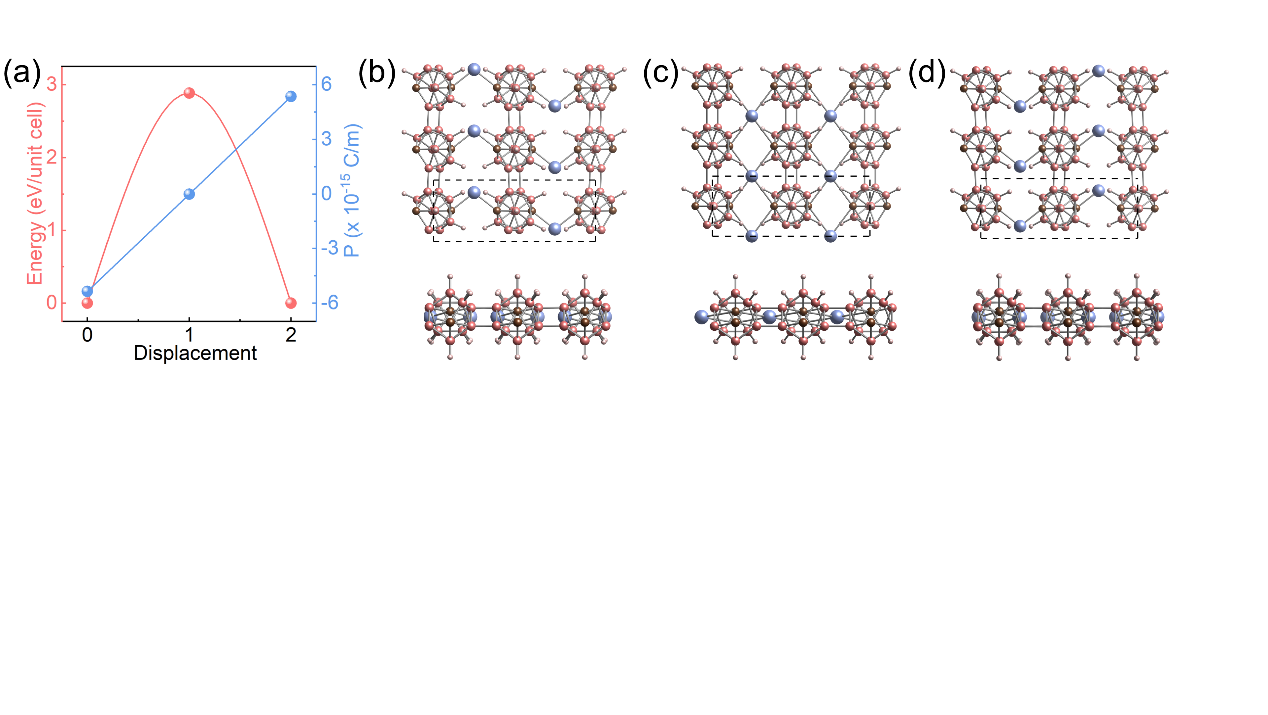


**Figure S13.** (a-d) Ferroelectric switching pathways and energy barrier for the NbB_10_C_2_H_6_ sheet.


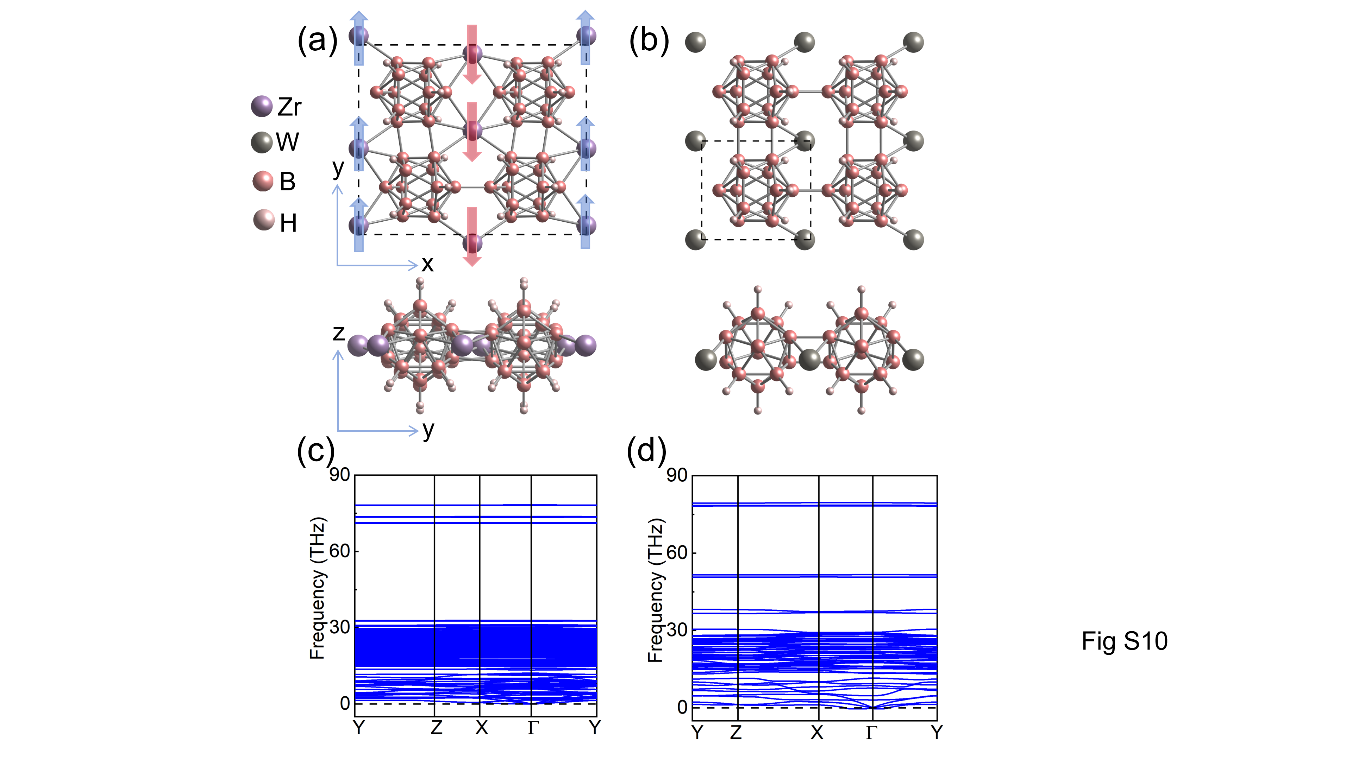


**Figure S14.** Top and side views of the (a) ZrB_12_H_6_ and (b) WB_12_H_6_ monolayer. The phonon dispersions of the (c) ZrB_12_H_6_ and (d) WB_12_H_6_.


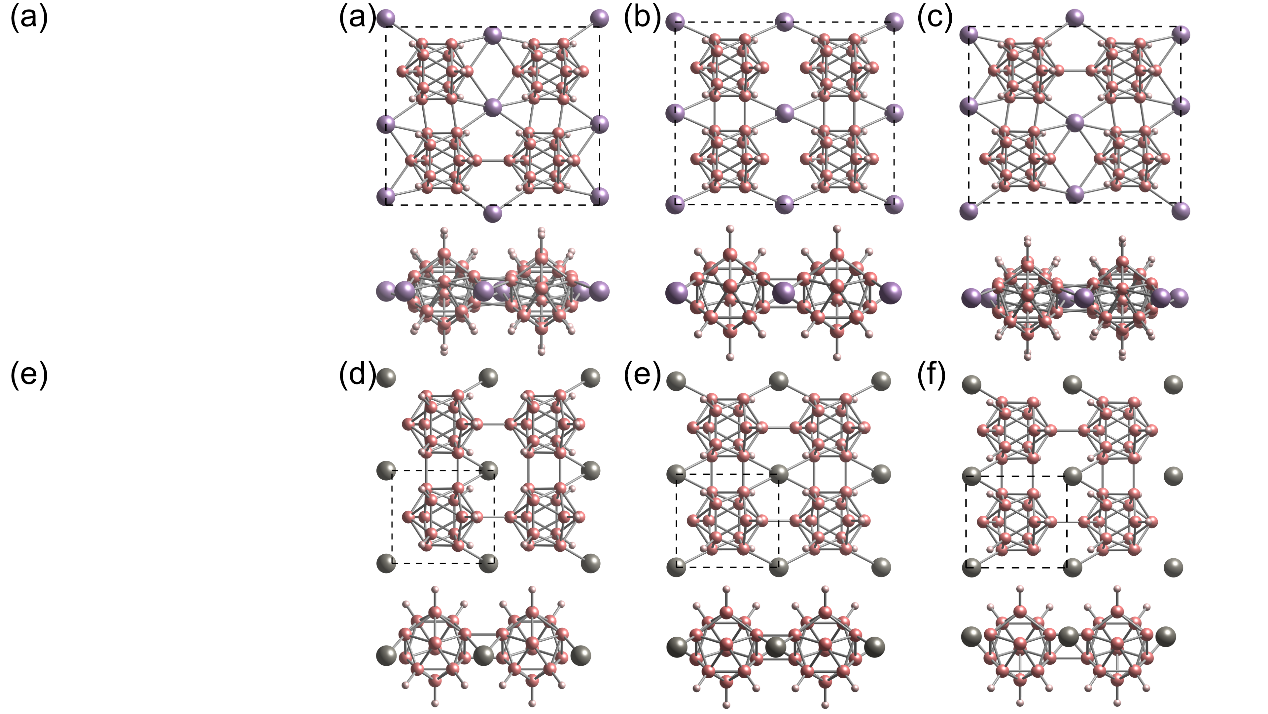


**Figure S15.** Ferroelectric switching pathways for the (a-c) ZrB_12_H_6_ and (d-f) WB_12_H_6_ monolayers.


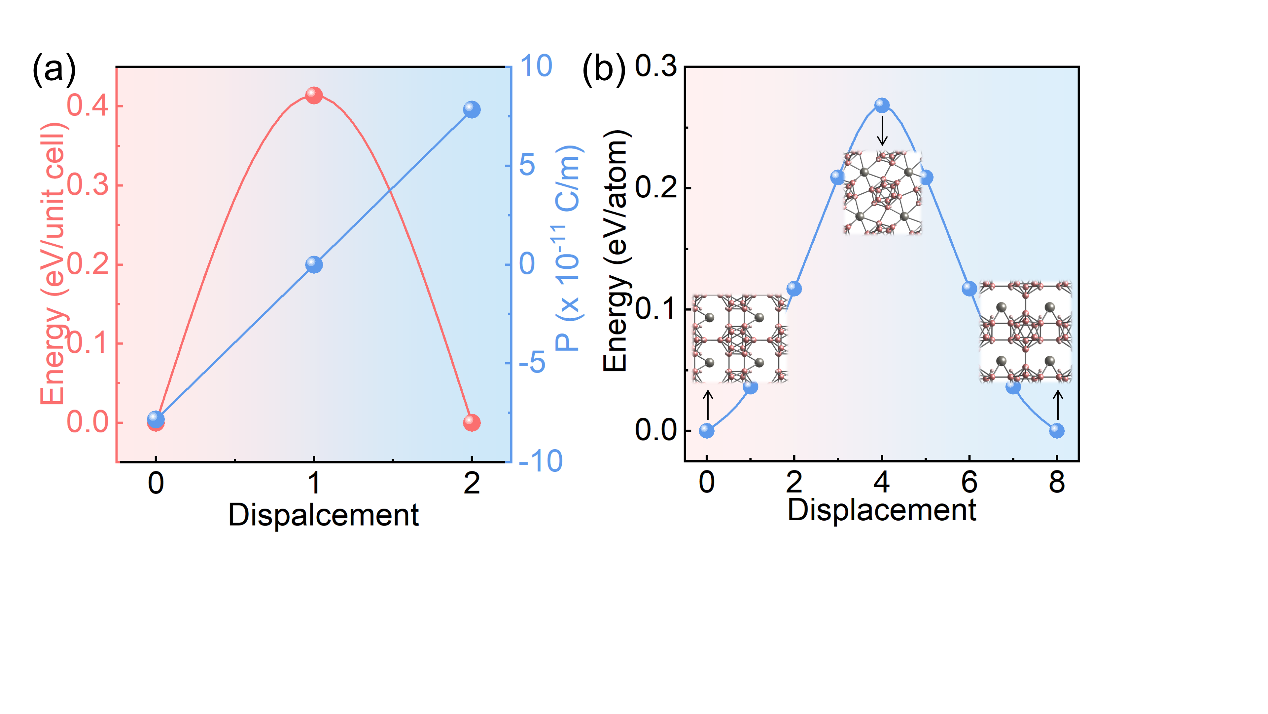


**Figure S16.** (a) Ferroelectric switching pathways and associated energy barriers for the WB_12_H_6_, yielding a spontaneous polarization of 7.83 × 10^-11^ C/m and a switching barrier of 0.44 eV per unit cell. (b) Ferroelastic switching pathways and corresponding energy barriers for the WB_12_H_6_, with a ferroelastic barrier of 0.27 eV per atom.


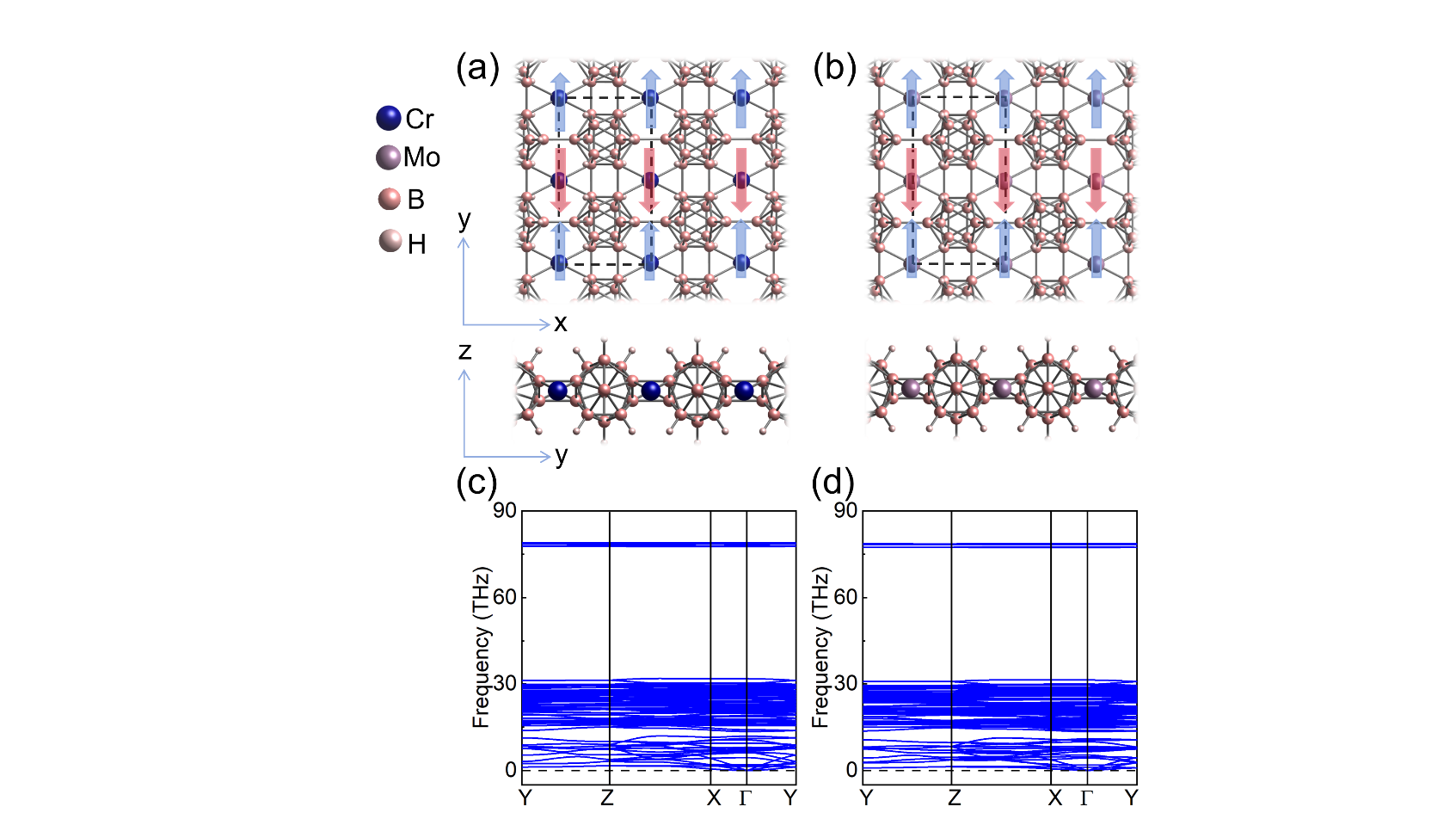


**Figure S17.** Top and side views of the (a) CrB_12_H_6_ and (b) MoB_12_H_6_ monolayer. The phonon dispersions of the (c) CrB_12_H_6_ and (d) MoB_12_H_6_.


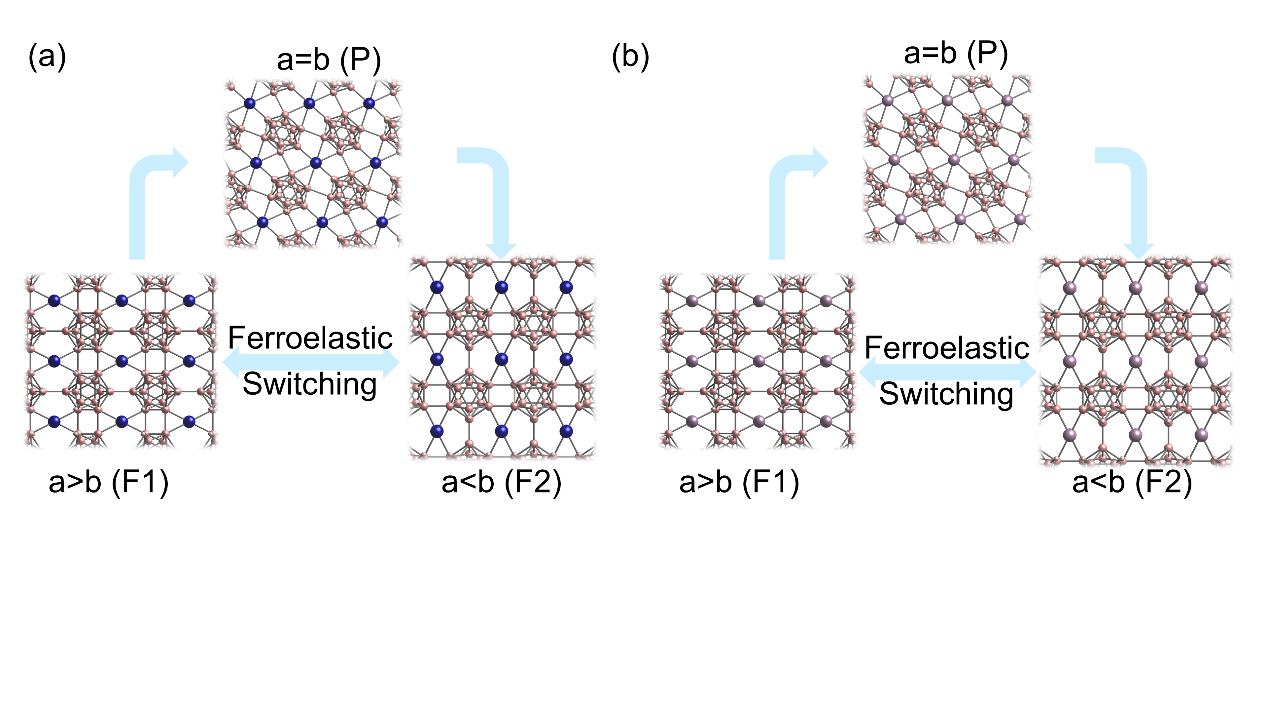


**Figure S18.** Ferroelastic switching among three ferroelastic variants for the (a) CrB_12_H_6_ and (b) MoB_12_H_6_.


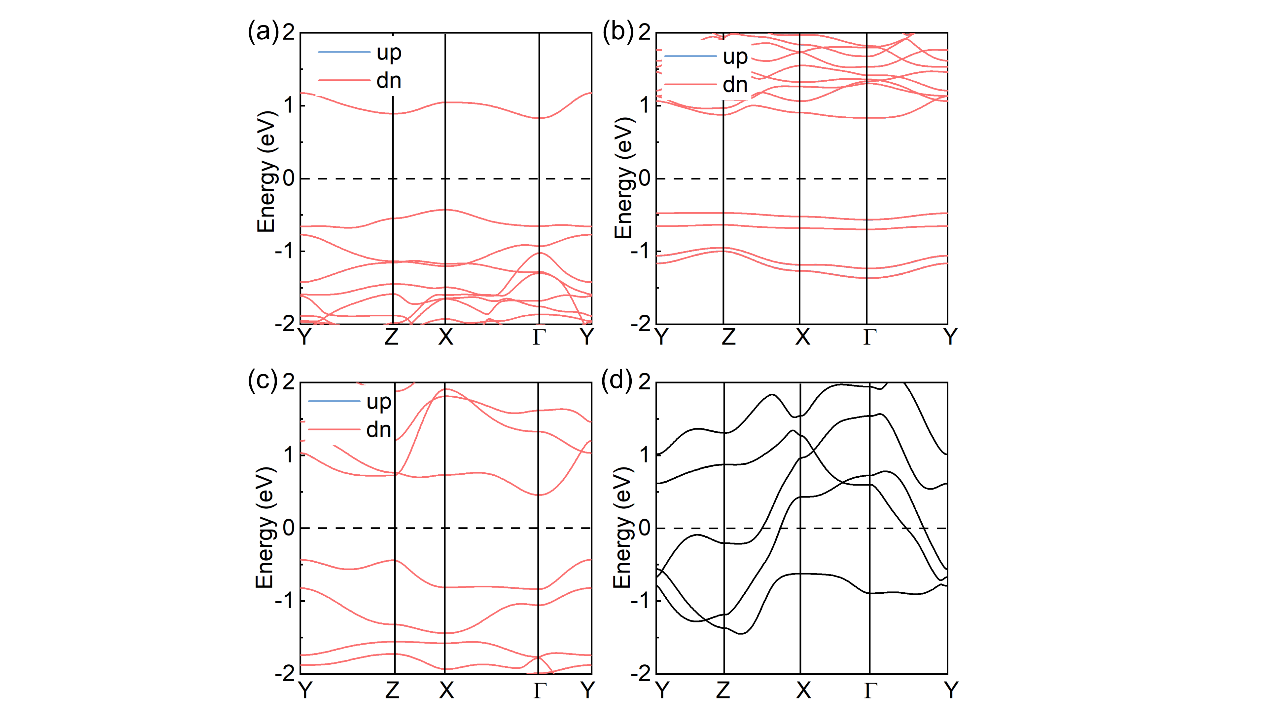


**Figure S19.** Band structure of (a) CrB_12_H_6_, (b) ZrB_12_H_6_, (c) MoB_12_H_6_ and (d) WB_12_H_6_ monolayers.

# **Bader charge analysis of the distorted boron cage**

**Table S1.** Electron gain (Q) of the boron cage (shown in Figure S3) based on Bader charge analysis.

|  | *Q* |
| --- | --- |
| B1 | 0.20 |
| B2 | 0.46 |
| B3 | 0.11 |
| B4 | 0.15 |
| B5 | 0.16 |
| B6 | 0.09 |

# **The elastic constants of NbB_12_H_6_ monolayer**

**Table S2.** The elastic constants (in GPa∙ nm) of NbB_12_H_6_ monolayer.

|  | C_11_ | C_12_ | C_22_ | C_66_ |
| --- | --- | --- | --- | --- |
| NbB_12_H_6_ | 173.20 | 36.48 | 178.41 | 25.86 |

**References**

[1] Z.-j. Wu, E.-j. Zhao, H.-p. Xiang, X.-f. Hao, X.-j. Liu, J. Meng, *Phys. Rev. B.* **2007**, *76*, 054115.

[2] K. Wang, T. Hu, F. Jia, G. Zhao, Y. Liu, I. V. Solovyev, A. P. Pyatakov, A. K. Zvezdin, W. Ren, *Appl. Phys. Lett.* **2019**, *114*, 092405.

[3] Y. Wang, J. Xing, Y. Zhao, Y. Wang, J. Zhao, X. Jiang, *Adv. Sci.* **2024**, *11*, 2401048.

[4] S.-S. Wang, Z.-M. Yu, Y. Liu, Y. Jiao, S. Guan, X.-L. Sheng, S. A. Yang, *Phys. Rev. Mater.* **2019**, *3*, 084201.

[5] Y. Che, H. Lv, X. Wu, J. Yang, *Chem. Sci.* **2024**, *15*, 13853.
